# Bio-mediated synthesis of 5-FU based nanoparticles employing orange fruit juice: a novel drug delivery system to treat skin fibrosarcoma in model animals

**DOI:** 10.1038/s41598-019-48180-7

**Published:** 2019-08-23

**Authors:** Owais Mohammad, Syed Mohd. Faisal, Nadeem Ahmad, Mohd. Ahmar Rauf, Mohd Saad Umar, Anzar Abdul Mujeeb, Piyush Pachauri, Anees Ahmed, Mohammad Kashif, Mohammad Ajmal, Swaleha Zubair

**Affiliations:** 1 Interdisciplinary Biotechnology Unit, AMU, Aligarh, India; 2 University of Jeddah, Jeddah, Saudi Arabia; 3 National Institute of Immunology, New Delhi, India; 4 Jawaharlal Nehru Medical College, AMU, Aligarh, India; 5 Department of Computer Science, AMU, Aligarh, India; 6 Women’s College, AMU, Aligarh, India

**Keywords:** Cancer, Oncology

## Abstract

Nano-sized drug delivery systems (NDDS) have been widely exploited to achieve targeted delivery of pharmaco-materials. Traditional pharmaceutical approaches, implied in the synthesis of nano-formulations, are obscure owing to the incompatible physico-chemical properties of the core drug as well as some other factors crucial in development of NDDS. Infact, most of the existing methods used in development of NDDS rely on usage of additives or excipients, a special class of chemicals. Barring few exceptions, the usage of synthetic excipients ought to be curtailed because of several associated undesirable features. Such issues necessitate strategies that lead to development of the synthetic excipient free drug delivery system. Plant based extracts have great potential to induce synthesis of nano-sized particles. Considering this fact, here we propose a prototype employing orange fruit juice (OJ) to facilitate bio-mediated synthesis of nano-sized supra-molecular assemblies of 5-fluorouracil (5-FU), a potent anticancer drug. The as-synthesized 5-FU Nanoparticles (NPs) retained the anti-neoplastic efficacy of the parent compound and induced apoptosis in cancer cells. The novel 5-FU NPs formulation demonstrated enhanced efficacy against DMBA induced experimental fibrosarcoma in the mouse model when compared to the micro-sized crystals of parent 5-FU drug.

## Introduction

Recent progression in the field of nanotechnology has made remarkable impact on human health and safety. Several recent reports have described a direct correlation between nanoparticles (NPs) size and higher efficacy of the associated drug^1,2^. Nanoparticle based drug delivery vehicles have been reported to preferentially accumulate at the site of injury, infection and inflammation, mostly because of endothelial dysfunction and blood vessel fenestration at such locations. The nano-dimensions of the NPs promote their ability to traverse various parts of the body. In general, NPs have tendency to accumulate into liver or spleen (components of reticulo-endothelial system) as well as other vital organs including lungs and brain^1–3^. Surface modification of NPs with specific ligands such as antibodies as well as aptamers *etc*. can facilitate their homing at desired site inside the host body. Upon gaining access to a specific biological niche, the nano-sized materials tend to interfere with a variety of cellular functions including cell proliferation, cell cycle regulation and various vital metabolic activities, *etc*.

5-Fluorouracil (5-FU), is an anti-metabolite showing broad spectrum anti-cancer activity against solid tumors^4^. Primarily, a pyrimidine analog, 5-FU is a purely ‘S-phase’ active chemotherapeutic agent (with no activity when cells are in G0 and G1 phase of cell cycle) that acts as a thymidylate synthase inhibitor and thereby interferes with DNA synthesis^5^. However, 5-FU drug has several limitations when used in clinical setting that include its short biological half-life, wide systemic distribution and marked toxic effects on bone marrow^6^. Numerous attempts have been made to reduce 5-FU associated side effects and eventually to enhance its therapeutic potential.

The efficacy of a therapeutic agent depends on its bioavailability that in turn is regulated by pharmacokinetic parameters of the related dosage form. Interestingly, various size associated unique features, associated with NPs of a specific chemotherapeutic agents, offers an extra edge over its free form in exerting a chemotherapeutic effect against a cancer cell population^7,8^. Unfortunately, in order to develop an effective nanosized delivery system, usage of synthetic excipients; the additives other than active pharmaceutical ingredient of a given drug formulation, is restricted mostly because some of them do possess various detrimental features such as undesirable biodegradability, short plasma half-life, toxicity and activation of host immune response, among others. Moreover, cost related issues of some of the widely used excipients, such as lipids in liposome, also hamper their wider application.

Bio-mediated synthesis of metal and inorganic compound based NPs employing extracts both from single and multi-cellular organisms has been considered as an enticing approach worldwide^9–11^. Recently available nano-sized drug materials, the so-called self-assembled metal NPs, synthesized by employing plant or microbial extract, have elicited enormous interest in nano-therapeutics^12–16^. Amazingly, these methods have not been translated into the synthesis of NPs comprising of therapeutically active organic molecules; with the exception of few reports from our group highlighting *Aloe vera* leaf extract mediated fabrication of nano-sized particles or assemblies^17^.

It is tempting to speculate that bio-mediated fabrication of 5-FU nano-assemblages employing orange fruit juice (OJ) offers a promising approach in the development of novel class of anticancer drug delivery system. Taking this fact into consideration, we explored OJ for its potential to induce the formation of nano-sized particles from microcrystals of parent 5-FU drug. The crystal habit and size analysis of the as-synthesized 5-FU NPs was characterized by employing transmission electron microscopy (TEM), Nanophox Analyzer, DLS, Zetasizer, and Atomic Force Microscopic studies.

To ascertain slow and sustained release of the parent 5-FU nucleobase, we examined the release kinetics of 5-FU NPs over an extended time period. The anti-cancer potential of as-synthesized 5-FU NPs was established by analyzing the level of various apoptotic markers in both epidermoid cell line (A253) as well as *in vivo* system. Finally, we evaluated the efficacy of as-synthesized 5-FU NPs in treatment of dimethyl-benz-α-anthracene (DMBA) induced fibrosarcoma in BALB/c mice.

## Results

### Content of orange juice

In the present study, we evaluated the potential of the OJ to facilitate bioconversion of 5-FU micro-sized crystals, into nano-sized tiny particles. Consistent with our earlier finding involving *Aloe vera* leaf extract mediated synthesis of NPs^17,18^, we found that incubation of 5-FU with OJ leads to the bio-mediated synthesis of 5-FU NPs. Data of the present study establish that newly formed NPs can inhibit cancer cells both *in vitro* as well as in model animals more efficiently when compared to the microcrystals of parent 5-FU drug. The detailed description of the orange juice ingredients and its other constituents is represented in Table. 1 and in Supplementary Figs S2 and S3.

**Table 1:** Various constituents of Orange Juice and its effects on the progression of cancer.

Ingredients of Orange Juice	Activity	Target and Mechanism of action
Naringenin	Anti-inflammatory	Down regulated ROS production by effecting the NF-κB via EGFR-PI3K-Akt/ERK MAP Kinase signalling pathway^47^.
Hesperetin	Anti-carcinogenic	Induced cytotoxicity in various cancers by down-regulating Bcl-2 expression and enhanced expression of Bax and Caspase-3^48^.
Hydroxycinnamic acid derivatives (Flavones)	Anti-oxidant	They have a potent free radical scavenger ability^40^.
D-Limonene (Limonoids)	Anti-proliferative	Suppressed Bcl-2 expression level, upregulated Bax level while caspase-9 was activated and inhibited the Akt activation via intrinsic mitochondrial apoptosis signaling pathway^49^.
Ascorbic acid (Vit C)	Anti-oxidant	Highly antioxidant power to recycle vitamin E in membrane and lipoprotein lipid peroxidation^50^.
Coumarins and Bioactive amines	Anti-carcinogenic and anti-thrombotic activities	Effect various pathways leading to cancer development such as kinase inhibition, cell cycle arrest, telomerase inhibition, antimitotic activity, carbonic anhydrase inhibition^51^.
Folate	DNA synthesis, repair and methylation	It modulates the risk of developing cancer in various tissues^52^

### Physico-chemical properties of 5-FU NPs

**Figure 1:**
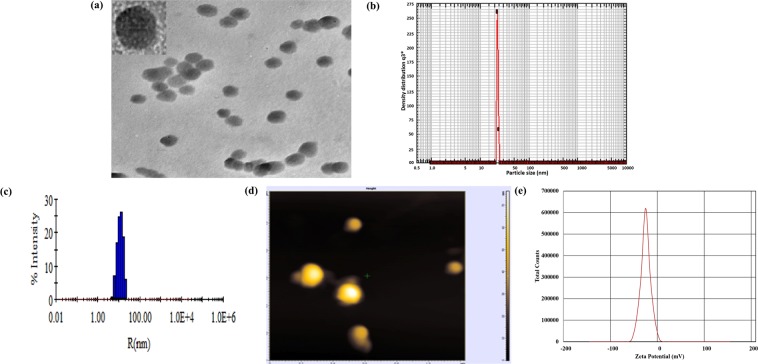
Characterization of 5-FU NPs employing TEM, HRTEM, Nanophox Particle Sizing, DLS, AFM and Zeta-potential analyses. (**a**) TEM analysis of 5-FU NPs synthesized by incubating 5 ml of 10^−3^ M 5-FU solution with 5 ml of OJ for 48 h. HRTEM image of the hexagonal 5-FU NPs (inset **1a**). (**b**) Corresponding particle analysis data as obtained by Nanophox particle analyzer, confirmed the average particle size of 5-FU NPs to be around 25 nm as evident from the single prominent peak (**c**) As analysed by Dynamic Light Scattering size distribution profile,the average size of 5-FU NPs was found to be 25 nm (**d**) Representative 2D Image of 5-FU NPs as revealed by Atomic Force Microscopy (**e**) Zeta potential of 5-FU NPs was found to be −39.6 ± 2.10 mv as revealed by Zetasizer analysis. The dialyzed OJ (2 Kd membrane cut off) failed to induce 5-FU nanoparticle formation. Treatment with proteinase, DNAse or RNAse did not affect potential of OJ to induce 5-FU NPs.

Figure. 1 illustrates characterization of the synthesized 5-FU NPs. The TEM picture reveals formation of isomorphic hexagonal 5-FU NPs (Fig. 1a and Supplementary Fig. S1c). HRTEM image further highlights the hexagonal shape and size of the formed NPs (inset Fig. 1a). In contrast to role of reducing agents as well as other protein constituents to facilitate synthesis of metal nanoparticle, the obscure composition of OJ complicates the analysis and identification of active species that are responsible for nucleation and growth of organic molecules based NPs. While it is not clear what forces drive the formation of 5-FU based NPs, nevertheless, preliminary studies suggest that particle formation is a result of coalescence of core drug nuclei leading to particle growth. Earlier reports propose that low molecular weight hydrophilic constituents of plants were responsible for bio-mediated synthesis of metal NPs^16,18^. In order to determine the role of low molecular weight compounds in inducing 5-FU NPs formation, the OJ was dialyzed through a 3 kDa cut off dialysis membrane. We found that the dialyzed extract lost its potential to induce NP synthesis.

We next ruled out nucleic acid or protein-based substances present in OJ for their putative role in NP formation. For this, OJ was treated with Proteinase K, DNAse or RNAse prior to its use in synthesis of 5-FU NPs. It was observed that the above specified treatment does not affect the ability of the OJ to facilitate the synthesis of 5-FU NPs (Fig. 1). To further rule out involvement of other protein based components, we precipitated the OJ with 80% ammonium sulfate and found that the supernatant left after complete precipitation was still efficient in facilitating 5-FU NP formation (data not shown). We also found that boiling of the OJ for 15 minutes did not alter its potential to form 5-FU NPs, which yet again indirectly rules out the involvement of protein based materials in induction of NPs formation.

In concordance with TEM analysis, particle size determinations employing Nanophox further confirmed the size of 5-FU NPs to be around 30 nm as evident from the single prominent peak in Figure 1b. The size distribution profile, as determined by Dynamic light scattering (DLS), also revealed that the NPs have an average size of 30 ± 5 nm as shown in Fig. 1c. Upon incubation of 5 ml of 10^−3^ M 5-FU with 5 ml of 30% w/v of OJ stock (stock solution: 30 g pulp in 100 ml of water) of OJ showed spontaneous synthesis of 5-FU NPs. The synthesis of a myriad of hexagonal shaped NPs was observed in 2D AFM microscopy as shown in Fig. 1d and Supplementary Fig. S1d. The 3D AFM image of as-synthesized 5-FU NPs has been depicted in the Supplementary File (Fig. S1e). All the employed studies unequivocally established size dimensions of the synthesized particles in the range of 25–35 nm. Zeta potential of the 5-FU NPs was found to be around −39.6 ± 2.10 mV as revealed by Zetasizer Nano potential analyzer. The high negative zeta potential value conferred stability to NPs and prevents their aggregation (Fig. 1e).

### Release kinetics of 5-FU NPs

**Figure 2:**
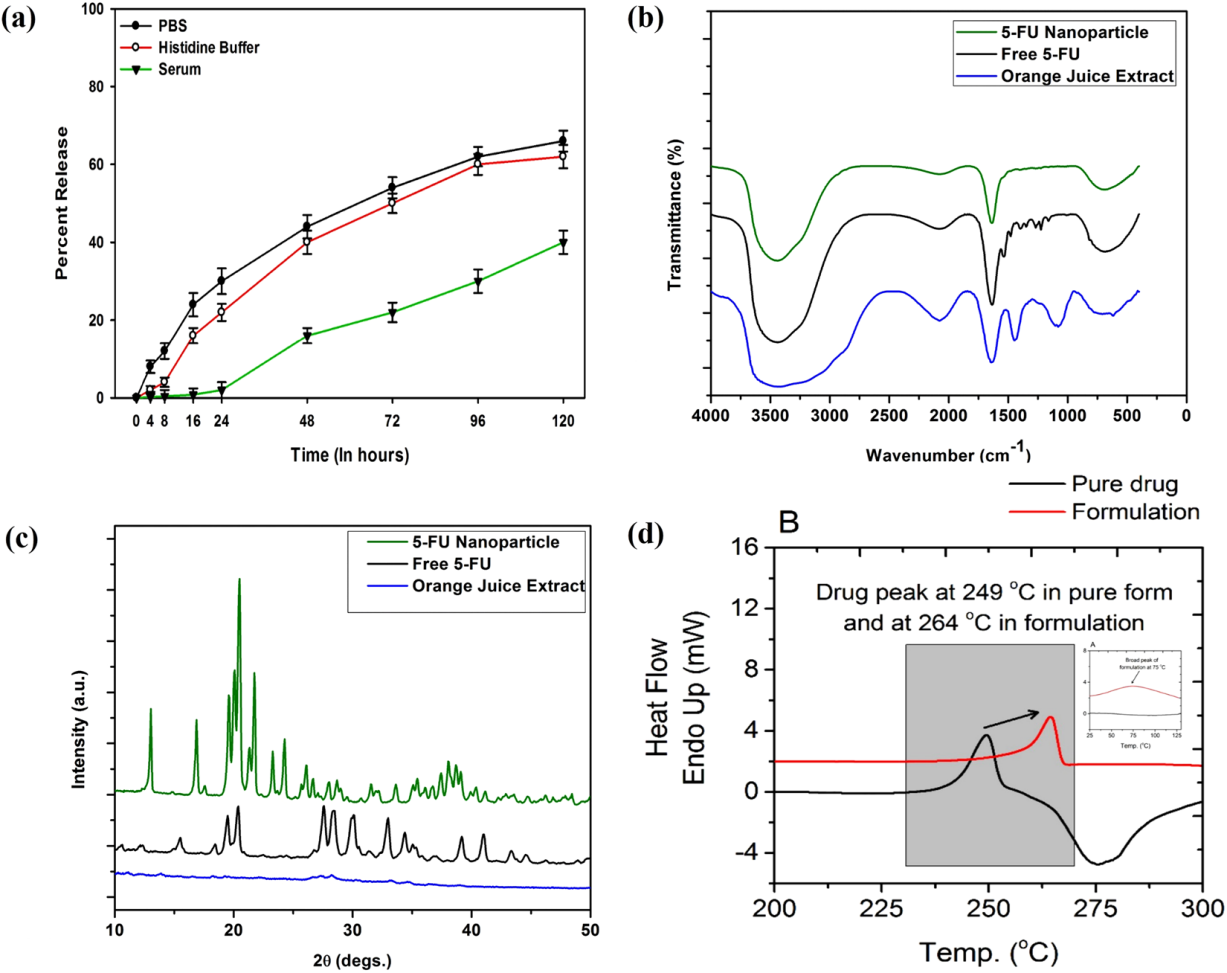
Assessment of purity and stability of as-synthesized 5-FU NPs employing FTIR, DSC, XRD analyses; To analyze stability of 5-FU NPs, multiple samples of the formulation were dispensed into various micro vials. After stipulated time period, the suspension was centrifuged and an aliquot of supernatant was analyzed to determine release pattern of the parent 5-FU molecules in the medium as described in Materials and Methods section; (**a**) stability of as-synthesized 5-FU NPs was determined by following release kinetics in PBS, histidine buffer as well as serum as incubation medium, (**b**) FTIR spectra, (**c**) XRD pattern and, (**d**) DSC thermograph of 5-FU NPs as determined by following method as described in ‘Methods’.

To establish that 5-FU NPs can work as a depot system of the parent drug, we performed a release kinetic study. The release kinetics data indicate stability of the NPs under various experimental conditions. We found that NPs endure their entity for a time period of more than 120 hours and released approx., 60% of the total drug in PBS as well as Histidine medium. Around 40% of the original drug was released in the surrounding medium when 5-FU NPs were co-incubated with serum (Fig. 2a).

### FT-IR spectra of 5-FU NPs

The IR spectrum of an organic molecule deciphers its chemical entity in terms of various functional groups present. The IR spectra of 5-FU NPs showed characteristic peaks of the parent drug (Fig. 2b). The spectral region ranging from 400–600 showed bending of ring halogen bonds in aromatic fluoro compounds, a characteristic peak of fluoro group. The peaks at 1100 to 1700 cm^−1^ represented the stretching vibrations of the C=O, −COO−, −CN and C=C functional groups. There was an increase in the band width in the range 3300–3500 cm^−1^, characteristic for hydrogen-bonded molecules (−OH••••HO−). The formation of molecular pores in the as-synthesized 5-FU NPs could be related to the influence of the hydroxyl groups, where corresponding bands appear in the region of deformational vibrations of the C–O bond. The bands in the range of 1450–1100 cm-1 are associated with stretching vibrations (deformation of C–O and C–O–C bonds) as well as C–H vibrations.

The FTIR spectrum of 5-FU nanoparticle exhibits characteristics peaks that correspond well to the peaks of parent pure 5-FU micro-crystals of drug except for minor differences that can be correlated with local environment or ambiance generated upon formation of the nanoparticle. The splitting of the peak increases in the nanoparticle spectrum when compared to pure 5-FU micro-crystals. This is because of the orange juice ingredient might have interacted with parent 5-FU molecules during the formation/synthesis of the nano-particulate form of the 5-FU drug. An enhancement in the intensity of the peak in the nanoparticle as compared to pure 5-FU micro-crystals is again an indication of the incorporation of orange juice ingredients into the nanoparticle. The FTIR spectrum of 5-FU nanoparticle showed the presence of a peak at ~740 cm^−1^ corresponding to the C-H out of plane vibration of CF=CH in addition to the peaks observed for the nanoparticle. An enhancement in the intensity of the N-H bending at ~1655cm^−1^ and a shift of the O-H and N-H stretching and a shift from ~3400 cm^−1^ to ~3456 cm^−1^ is observed. All these observations confirm the presence of active component 5-FU in as-synthesized nanoparticles.

### XRD pattern and DSC analysis of 5-FU NPs

Figure 2c shows X-ray diffraction (XRD) patterns of both microcrystals as well as NP form of 5-FU. All the observed diffraction peaks can be indexed using standard data, which exhibits clear crystalline nature of as-synthesized NPs. Moreover, no impurity peaks were detected in the pattern of both forms of the drug, which suggest the high phase purity of the samples. The 2θ value of the most intense peak has been observed at ~ 37°.

Differential Scanning Calorimetry (DSC) is a thermoanalytical technique where the difference, between the amounts of heat required to increase the temperature of a sample and reference, is measured as a function of temperature and time^19,20^. Differential scanning calorimetry technique has been used previously for determination of crystallinity, purity, degradation and/or presence of drug in various pharmaceutical formulations as well^19,20^. The DSC thermogram suggests that microcrystals of precursor 5-FU drug show good thermal stability up to its melting point. DSC thermogram showed characteristic sharp peak of parent 5-FU microcrystal sample at around 235–255 °C with peak maximum at 249 °C (Fig. 2d). The thermogram corresponding to Nano-formulation, depicted two different peaks in the range of 25–130 °C and 240–270 °C with peak maxima at 74 °C (inset Fig. 2d) and 264 °C respectively. The broad peak corresponding to microcrystalline 5-FU changed significantly by ~15 °C in as-synthesized NPs. The observed change in thermal behaviour could be attributed to alteration in physical form of 5-FU in as-synthesized NPs. It also suggests higher stability of the formed NPs that may cause release of parent drug in slow and sustained manner.

The observed peak shift in DSC thermogram indicates that it requires a great amount of energy to break crystal structure of synthesized NPs when compared to microcrystals of parent 5-FU drug. Overall thermal analysis study suggests that the formed NPs are consisted of 5-FU mainly. The emergence of extra peak in thermogram suggests entanglement of 5-FU molecules associated with some putative components of OJ in the formed NPs^21^.

### Anticancer potential of as-synthesized 5-FU NPs

#### 5-FU NPs up-regulate Bax and p53wt expression in skin fibrosarcoma cells

We tried to unravel the pathway responsible for 5-FU NPs mediated modulation of both pro and anti-apoptotic proteins in epidermoid cells. 5-FU NPs significantly up-regulate expression of pro-apoptotic proteins p53 and Bax and down regulate Bcl-2 when compared to the parent drug. The enhanced expression of Bax can be attributed to the 5-FU NP mediated activation of mitochondria dependent apoptotic pathway. Reduction of mitochondrial membrane potential ensues in Cytochrome-C release that in turn leads to caspase activation^22^. The p53 protein induces expression of various subsets of genes leading to cell cycle arrest^23,24^. Further, it works as a transcription factor that interacts with a p53-specific DNA consensus sequence, required for the enhanced expression of p21 molecule^24–26^. The as-synthesized 5-FU NPs modulated both intrinsic as well as extrinsic pathways to induce apoptosis.

**Figure 3:**
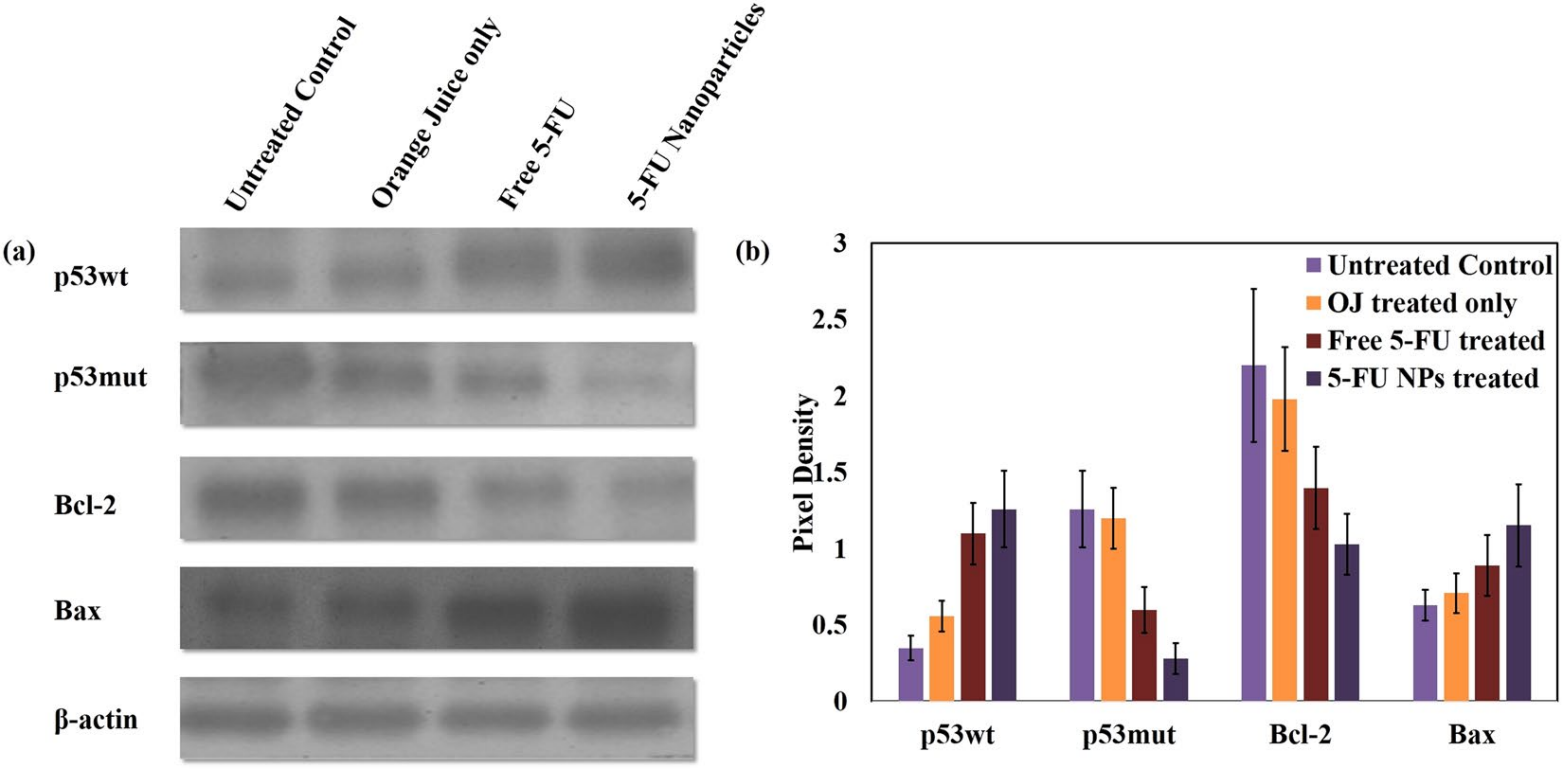
Effect of 5-FU NPs on expression of pro/anti apoptotic factors in tumor tissues from various experimental animal groups. (**a**) Cell lysates were prepared as described in Materials and Methods and analysed for expression of various proteins using specific antibodies. To ensure equal loading, the membranes were also probed with β-actin antibody. Densitograms show relative pixel density of p53wt, p53mut, Bcl-2 and Bax after treatment with various formulations as mentioned below. Lane 1, Untreated control (with cream base only); Lane 2, OJ along with cream base; Lane 3, Microcrystals of free 5-FU drug along with cream base; and Lane 4, 5-FU NPs prepared by mixing of 5-FU (10^−3^ M) solution with 5 ml of OJ (along with cream base). All the SDS-PAGE gels were run under similar experimental conditions. (**b**) Representative densitometry graph of the western blot. Pixel density of each band was quantified by using GS-800 Calibrated Imaging Densitometer. Statistical significance was calculated using unpaired student’s t-test. p ≤ 0.05 is considered statistically significant (Free 5-FU vs 5-FU NPs p < 0.05, Free 5-FU vs untreated control p < 0.01 and 5-FU NPs vs untreated control p < 0.001). Data represented as mean ± Standard Deviation

After establishing operative apoptotic pathway in epidermoid cancer cell line, next we elucidated anticancer potential of 5-FU NPs against skin fibrosarcoma in animal model. Animals, with established skin fibrosarcoma (DMBA-induced), were randomly divided in to four different groups and treated with various 5-FU formulations. Animals treated with 5-FU NPs demonstrated an elevated expression of p53wt as compared to those treated with the free form of the parent 5-FU drug (**p < 0.01) (Fig. 3). Moreover, 5-FU NPs strongly down-regulated expression of p53mut (p < 0.01), thereby enhancing apoptosis of cancer cells in model animals. The expression of pro-apoptotic and anti-apoptotic proteins was not significantly altered in control groups (animal groups treated with cream base or OJ), this proposes that regulation of p53mut gene expression is not because of orange fruit juice constituents or the cream base used as a carrier.

**Figure 4:**
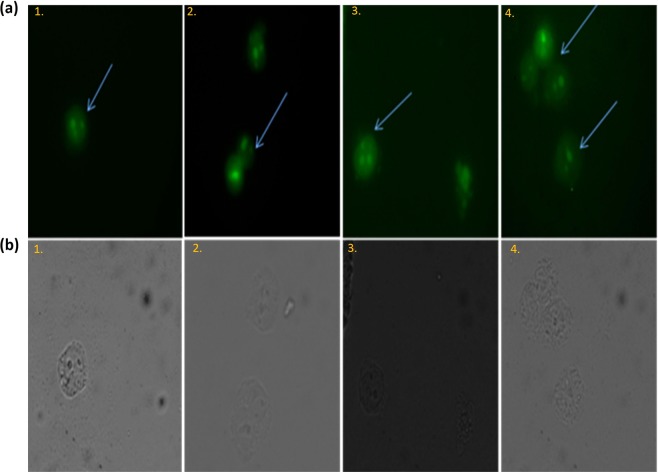
5-FU NPs treatment results in Caspase-9 mediated apoptosis of fibrosarcoma cells as revealed by fluorescence microscopy. (**a**) Fluorescence micrographs (**b**) Phase contrast micrographs. Untreated group served as control (panel 1), the other experimental groups are: (panel 2) OJ with cream base, (panel 3) Microcrystals of free 5-FU drug with cream base, and (panel 4) 5-FU NPs with cream base. Cells obtained from the various experimental groups were visualized under a fluorescence microscope at 40X magnification (excitation at 488 nm, emission at 505–530 nm).

Intrinsic pathway mediated release of mitochondrial cytochrome-C switches on caspase9/apoptosome to activate effector caspase-3, which cleaves many of the major constituents of the cell cytoskeleton and is important for the programmed dismantling of vital cellular structures^27,28^. Considering, caspases are canonically pivotal mediators of apoptosis, we determined the expression level of caspase-9 in various treated groups. Results shown in Fig. 4 demonstrate significantly enhanced expression of caspase-9 in 5-FU NPs treated group as compared to the group that received microcrystals of free 5-FU drug only (p < 0.01). There was relatively lower expression of caspase-9 in OJ (alone) as well as cream base only treated control groups. The data suggests that 5-FU NPs demonstrate remarkable efficacy in up-regulation of caspase-9 and facilitating apoptosis of fibrosarcoma in mouse model.

One can argue that cream base used for ease and presentation of as-synthesized 5-FU NPs can alter anticancer potential of the formulation. To rule out this possibility we applied 5-FU NPs suspended in normal saline on the fibrosarcoma harboring animals. We found that 5-FU NPs suspended in normal saline, in a manner similar to 5 FU-NPs suspended in cream base, showed same degree of fibrosarcoma regression as well as modulation of various factors viz. p53wt, Bax and Bcl2 in Balb/c mice (Fig. S5).

#### 5-FU NPs mediated DNA fragmentation

**Figure 5:**
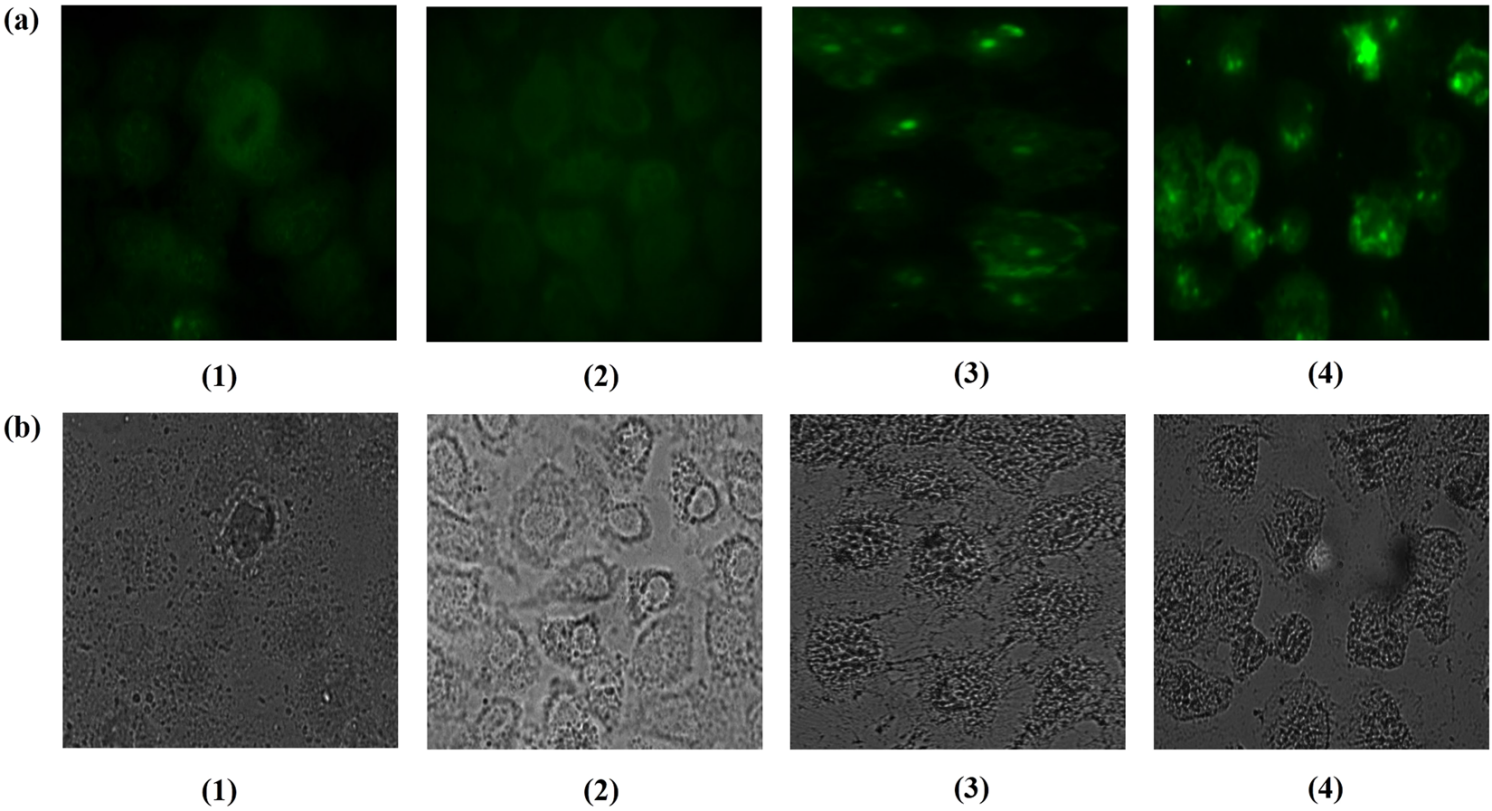
5-FU NPs induce enhanced apoptosis potential in the treated animals as revealed by DNA fragmentation. (**a**) Fluorescence micrographs (**b**) Phase contrast. Nanoparticle mediated DNA fragmentation as observed by Apo-BRDU analysis by employing fluorescence microscopy. 5-FU NPs treated cells harbour large numbers of DNA breaks when compared with the parent 5-FU drug. While group (1) served as control (untreated), the other treated groups are: (2) OJ with cream base, (3) Microcrystals of free 5-FU drug with cream base, and (4) 5-FU NPs with cream base. Cells obtained from the various experimental groups were scanned under a fluorescence microscope at 40X magnification (excitation at 488 nm, emission at 505–530 nm).

5-FU NPs mediated activation of endo-nucleases, during the apoptosis, ensues in DNA fragmentation. The nucleases break the higher order structure of chromatin into smaller fragments and finally into smaller DNA strands. Efficacy of 5-FU NPs was observed by BrdU tunnel analysis using FACS and fluorescence microscopy. The enzyme TdT catalyzes a template dependent addition of Br-dUTP to 3-hydroxyl ends of double and single stranded DNA. After incorporation, DNA breaks were identified by a FITC-labeled anti-BrdU mAb. Apoptosis induction was found to be significantly enhanced (***p < 0.001) in 5-FU NPs treated animals (Fig. 5a,b) as compared to the group treated with microcrystals of free 5-FU drug (*p < 0.05) (Fig. 5a,b), while both “OJ only in cream base” treated group as well as ‘cream base only treated control group’ showed fewer number of fluorescent labelled cells as revealed in Fig. 5a.

**Figure 6:**
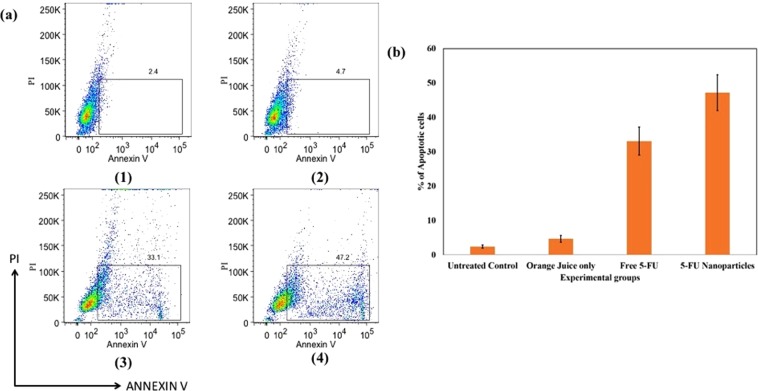
Effects of as-synthesized 5-FU NPs on the induction of apoptosis as analysed by Annexin V binding study. (**a**) Annexin V apoptosis detection employing FACS analysis. Apoptosis induction was assessed in the cells isolated from various experimental groups. As revealed by Annexin V binding, apoptosis is significantly augmented in cells belonging to 5-FU NPs treated mice when compared to microcrystals of free 5-FU drug treated mice. While group (1) served as control (untreated), the other treated groups are: (2) OJ with cream base, (3) Microcrystals of free 5-FU drug with cream base, and (4) 5-FU NPs with cream base. (**b**)The percentage of apoptotic cells was significantly enhanced in the 5-FU NPs (5 FU-NPs vs untreated control p ≤ 0.001) treated group of animals when compared to parent 5-FU drug (Free 5 FU vs untreated control p ≤ 0.05) as represented in the form of Histogram.

Apoptosis mediated loss of phospholipid asymmetry in the plasma membrane was observed using FITC Annexin-V staining. Apoptosis is significantly augmented in cells representing the 5-FU NPs treated group (Fig. 6a,b) when compared to that of microcrystals of free 5-FU drug treated animals (Fig. 6a). There was no significant augmentation of apoptosis in cells obtained from cream base treated mice as well as from the OJ along with cream base treated group (Fig. 6a), respectively).

#### Anticancer efficacy of 5-FU NPs against DMBA-induced fibrosarcoma

**Figure 7:**
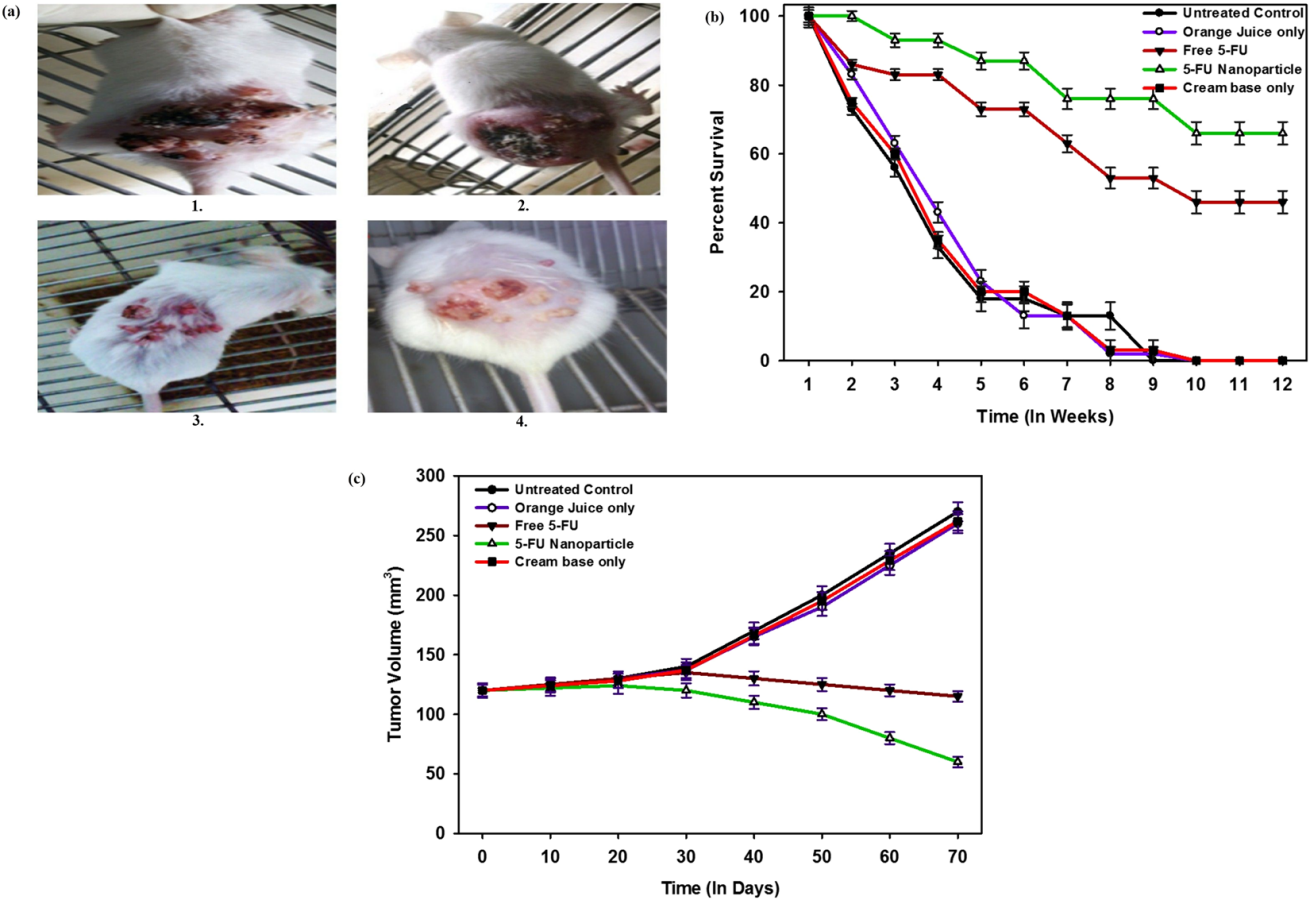
Efficacy of the as-synthesized 5-FU NPs as revealed by survival, regression in tumor volume and fibrosarcoma suppression. (**a**) 5-FU NPs mediated supression of DMBA induced fibrosarcoma in *BALB/c* mice. *BALB/c* mice were exposed to DMBA to induce fibrosarcoma following method as described in methodology section. The animals with fibrosarcoma were divided in four groups viz. Group (1) represent untreated control animals while other groups are (2) OJ along with cream base, (3) Microcrystals of free 5-FU drug along with cream base, (4) 5-FU NPs along with cream base, (**b**) Effect of various formulation on survival of *BALB/c* mice with fibrosarcoma. Survival data depicting efficacy of 5-FU NPs formulation in terms of percent survival at various time points (post treatment). While group (1) served as control (untreated), the other treated groups were: (2) OJ with cream base, (3) Microcrystals of free 5-FU drug with cream base, and (4) 5-FU NPs with cream base. The animals treated with 5-FU NPs showed significantly better survival (p < 0.001) as compared to control groups. p value < 0.001; 5FU-NPs vs untreated control and p value < 0.01, free 5-FU(microcrystalline) vs untreated control group. (**c**) Tumor voulme regression in animals with fibrosarcoma after treatment with various formulations of 5-FU drug. p value ≤ 0.01; 5-FU NPs vs untreated group.

Kaplan-Mier Curve for survival analysis was used to evaluate the efficacy of as-synthesized 5-FU NPs against DMBA-induced fibrosarcoma in model animals (Fig. 7b). The survival of mice inflicted with fibrosarcoma was determined at various time intervals. Treatment with cream base alone or OJ in combination with cream base was not of much help as the animals did not survive for more than 8–9 weeks. On the other hand, survival rate at 12 weeks increased up to 45% in 5-FU microcrystals drug treated animals. Interestingly, 5-FU NPs treated animals showed dramatic increase in survival rate (68%) (***p < 0.001) when compared to control untreated group.

Effect of 5-FU NPs on the regression in tumor volume was also measured. The mean tumor volume per animal was significantly lower in animals treated with 5-FU NPs (40.4 mm^3^, p < 0.001) compared to those treated with the microcrystals form of drug (73.1 mm^3^, p < 0.001). On the other hand, treatment with a mixture of OJ in cream base and cream base alone (vehicle) resulted in much higher mean tumor volume compared to nano-assembled 5-FU drug (Fig. 7c).

**Figure 8:**
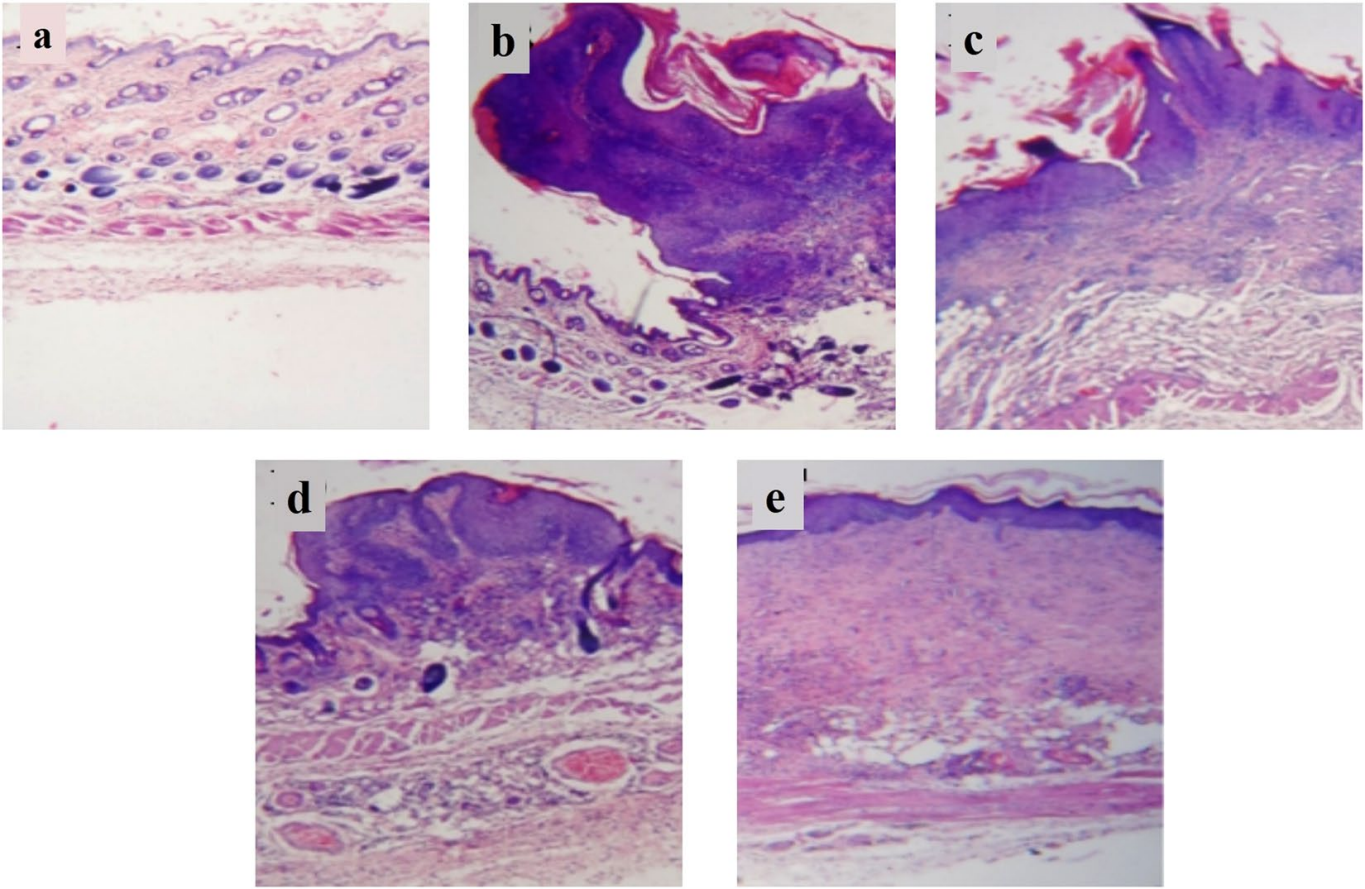
Histopathological analysis of excised tissue isolated from 5-FU NPstreated experimental animals. The histo-pathological results of the excised tissue from various treated experimental groups. (**a**) Healthy control animals showed normal skin with epidermal pegs, dermal papillae, healthy pilocebacious units with uniform thickness of epidermis and its associated keratinization, (**b**) Untreated animals with cancer or animals treated with cream base (carrier) only, (**c**) Animals treated with OJ along with cream base, (**d**) Animals treated with microcrystals of free 5-FU drug showed papillomatous change, heavy acanthosis as well as keratosis, and (**e**) Animals treated with Nano-assembled 5-FU NPs resulted in sessile to pedunculated papillomatous change with heavy acanthosis and marked keratosis.

The histo-pathological analysis of the excised tissue from various treated experimental groups further confirmed higher efficacy of 5-FU NPs. Healthy control animals showed normal skin with epidermal pegs, dermal papillae, and healthy pilosebaceous units with uniform thickness of epidermis and its associated keratinization (Fig. 8a). Untreated animals with cancer (Fig. 8b) showed papillary and cauliflowers like growth with remarkable increase in epidermal thickness and keratinization. Animals treated with cream base (carrier) or OJ along with cream base demonstrated papillomatous growth, moderate acanthosis and mild keratosis, complex fibro vascular core with congested vessels and loss of pilosebaceous units (Fig. 8c). Animals treated with microcrystals of free 5-FU drug showed papillomatous change, heavy acanthosis as well as keratosis (Fig. 8d). On the other hand, mice treated with 5-FU NPs resulted in sessile to pedunculated papillomatous change with heavy acanthosis and marked keratosis (Fig. 8e).

## Discussion

Among various novel drug delivery systems, NPs have emerged as a suitable drug vehicle in regulating pharmacokinetics, pharmacodynamics and eventually the bioactivity of the active core compound^1,28^. NPs entail enroute shielding/protection of the associated drug molecules and eventually facilitate their targeted delivery to the active site^1,28,29^. In spite of their widely acclaimed potential for sustained drug release and potential to accumulate at the desired site, NPs do come across with series of barriers that impede attainment of desirable therapeutic outcome^28–30^.

In the present study, we have established that orange juice extract (OJ) can induce formation of 5-FU NPs with exceptional physico-chemical attributes. The OJ constituents seems to regulate synthesis of as-synthesised 5-FU NPs in intriguing fashion. The incubation with relatively less OJ content (1 ml OJ with 5 ml of 10^−3^ M 5-FU solution; final volume of reaction mixture 10 ml) resulted in decrease in the absorbance. Incubation of drug with relatively larger ratio of OJ (3 ml OJ; 5 ml of 10^−3^ M 5-FU solution in final volume of the 10 ml) ensued in resurrection of characteristics 5-FU peaks. Further increase in OJ content (5 ml OJ; 5 ml of 10^−3^ M 5-FU solution keeping final volume upto 10 ml) resulted in restoration of the absorbance peak that tends to overlap with the peak height of the microcrystals of free 5-FU drug (data not shown). This clearly suggests that higher concentration of the active components of OJ facilitates ready formation of 5-FU NPs without changing the chemical structure of the parent nucleobase.

The UV absorption spectrum suggests that the microcrystals of free 5-FU drug absorbs maximally at 271 nm (Fig. S1d). Interaction of microcrystalline 5-FU with OJ resulted in significant quenching in absorbance of characteristic peaks belonging to 5-FU. Interestingly, longer incubation of 5-FU with OJ resulted in resurgence of absorbance with significant increase in intensity of various characteristic peaks in a time dependent manner. The resurrection of characteristic peaks with time suggests that chemical nature of 5-FU molecules is preserved during the synthesis of NPs. The significant higher absorption at ∼245 nm contributed by C=O chromophore remains unscathed during NP synthesis. (Fig. S1d). Similarly, absorption peak at ~192 nm, which is contributed by hetero atom (–N) containing pyrimidine ring, also remained intact in the as-formed 5-FU NPs. The blue shift of peak at 272 nm suggests that chromophore actively participate in non-covalent bonding of parent 5 FU molecules in as-synthesised NPs. The microenvironment of a chromophoric groups of a given chemical compound affects the Sπ life-span by tuning the relative energy of the Sπ state and the closely lying Sn dark state^31^. The stability of π-π* transition (in the case of 5-FU; it is the transfer of an electron from the lone pair of oxygen or nitrogen atom towards the more diffuse π* orbital) increases both with the polarity and hydrogen bonding ability of 5-FU with the external milieu. It seems that, the external milieu of an individual 5-FU molecule, in a given nanoparticle, modulates both π-π* as well as n-π* transitions^32,33^.

Dietary flavonoids specifically naringenin, hesperetin and quercetin *etc*.^34^ present in OJ have great potential in suppressing synthesis of DNA, RNA and proteins and demonstrate a detrimental effect on rapidly growing cells including cancerous cells. Interestingly, various secondary metabolites present in OJ have been categorically reported to possess strong anticancer potential against skin fibrosarcoma and colon carcinogenesis amongst others (Table 1).

One can argue that as-synthesized 5-FU NPs may contain OJ components incorporated during their synthesis. Considering it a desirable feature, as OJ has been reported to contain plethora of antioxidant substances with anticancer potential, we performed FT-IR, DSC and XRD analysis of as-formed 5-FU NPs to elucidate this possibility. Incidentally, because of overlapping chemical characteristics of the 5-FU with that of OJ components (small molecules with aromatic nucleus), the FT-IR data fail to reveal much about presence of OJ components in the as-synthesized 5-FU NPs. Nevertheless, specific wave numbers corresponding to a given functional group moiety suggests presence of very small amount of OJ contents in as-formed NPs. In contrast, DSC analysis suggests that while 5-FU molecules form the core of as-synthesized NPs. There was an additional peak corresponding to much less temperature. It seems this peak refers to the energy needed to break bonding of 5-FU drug molecules (intra-particle) present in a given as-synthesized 5FU-NP, that remain associated with each other as well as with residual OJ constituents still present in the NPs, via non-covalent bonds.

It can be speculated that the residual OJ contents still present in 5-FU NPs may exert their anti-cancer effects and help in effective suppression of DMBA induced skin cancer in the experimental mice. In addition, the fabricated 5-FU NPs release the active drug for an extended time period. The particles remain intact even in the presence of plasma components and released around 60% of the entrapped drug in 5 days. It seems OJ mediated 5-FU NP synthesis progresses via a self-nucleation process initially that eventually ensues in formation of primary particles^32^. The as-formed primary nuclei function as a seed to facilitate particle growth accompanied by an increase in the thermodynamic stability. The synthesis of NPs further followed heterogeneous nucleation and growth; a process referred to as Ostwald ripening. In process of NP fabrication, higher oligomers trap the monomers in their native orientation and inhibit structural rearrangements^31–33^. It can, therefore, be stipulated that the 5-FU dimers/tetramers can act as seeding point (nucleus) for the condensation of monomers and eventually lead to the formation of super-aggregated NPs. The contents of OJ function both as a driving force as well as stabilizing agent in the whole process.

Nanoparticle based drug formulation occupies a special status as a means to increase the efficacy of parent drug molecules. In order to study the efficacy of synthesized 5-FU NPs we carried out various apoptosis related studies employing different *in vitro* assays on epidermoid cancer cell lines (A253) viz. MTT assay (Fig. S4A), Western blot analysis of cell cycle regulating proteins *etc*. (Fig. S4B). The *in vitro* data establishes higher efficacy of formed 5-FU NPs and also suggest that the observed anti-cancer effects are mediated by physical presence of NPs and not by the controlled delivery route that may result in a greater amount of free drug reaching the tumor cells (Fig. S4C to E).

The real anti-cancer potential of 5-FU NPs, described in the present study, cannot be achieved without performing *in-vivo* studies. Therefore, in the next set of experiments, the anticancer potential of 5-FU NPs was evaluated by analyzing the expression levels of various pro and anti-apoptotic factors in the fibrosarcoma skin tissues of model animals. p53, a tumor suppressor gene that integrates various signals that regulate cell cycle is manipulated in cancer cells^25,35^. The failure of p53 expression (*cf*. p53mut induction) generally ensues in tumor development. Interestingly, treatment of the model animals with as-synthesized 5-FU NPs was successful in restoring expression of p53 as compared to treatment with the free form of 5-FU drug (*p < 0.05). Intriguingly, the treatment with as-synthesized 5-FU NPs also augmented expression of Bax, a p53 target that Trans-activates numerous factors during p53-dependent apoptosis, in the fibrosarcoma tissue.

The high pro-apoptotic efficacy of as synthesized 5-FU NPs was also confirmed by analyzing expression level of p53mut and Bcl-2 in the cancerous tissues of the treated animals^22,24,36,37^. The 5-FU NPs markedly down regulate expression of both Bcl-2 and p53mut. Annexin V apoptosis analysis that detects the presence of phosphatidyl-serine in the outer leaflet of cancer cells further confirms apoptosis induction by treatment with 5-FU NPs.

The observed higher efficacy of 5-FU NPs can be correlated with better penetration and retention of 5-FU NPs as compared to parent 5-FU drug. Upon their application to the skin, the internalized NPs release their payload (active drug) into the cells that consequently forms the basis of better efficacy over the free form (microcrystalline)of the drug. The histopathological studies support the theory that 5-FU NPs facilitate efficient killing of cancer cells as compared to parent 5-FU drug (Fig. 8).

The excipient-free Nanoparticle based novel drug delivery system, introduced in the present study, holds great promise in release of the encore drug molecules in slow and sustained manner. This effectively influences the cell cycle check points, thereby causing effective killing of the cancer cells and significantly reduce the level of mortality in fibrosarcoma animals.

## Conclusions

The present study reports orange juice content mediated synthesis of 5-FU NPs. The biophysical characterization suggests synthesis of homogenous population of 5-FU NPs with nano-meter size dimensions. The 5-FU NPs were found to kill epidermoid cancer cells more efficiently as compared to the micro-sized crystals of 5-FU. Both i*n vitro* as well as *in vivo* studies proposed that 5-FU NPs modulate various components of cell cycle to induce apoptosis of target cancer cells. The as-synthesized NPs successfully increase efficacy of parent 5-FU drug in curing experimental murine fibrosarcoma in Balb/c mice.

## Methods

### Reagents

Reagents used in the experiments were of high purity.5-Fluorouracil, bicinchoninic acid (BCA), dialysis tubing cellulose membrane (cut off size MW = 3kD), syringe-driven filters of 0.22μm size were procured from HiMedia Laboratories Pvt. Ltd India. Antibodies for the apoptosis detection were purchased from BD Biosciences India and Santa Cruz biotechnology, Inc. Rabbit anti-mouse p53wt, rabbit anti-mouse p53mut, rabbit anti-mouse Bcl-2, rabbit anti-mouse Bax, rabbit anti-mouse β-actin, FITC labelled Annexin-V, caspase-9 p35 antibody and goat anti-rabbit IgG-FITC conjugated secondary antibody were the product of Sigma-Aldrich, USA.

### Ethical conduct of research

Inbred female BALB/c mice (6–8 weeks old, 20 ± 2 g) were obtained from the Animal House Facility of Interdisciplinary Biotechnology Unit, Aligarh Muslim University. The BALB/c mice were housed in commercially available polypropylene cages and maintained under controlled temperature conditions on a 12 hr light-dark cycle and had free access to food and water ad libitum. All experimental procedures involving animals were approved by the Institutional Animal Ethics Committee (IAEC) of the Interdisciplinary Biotechnology Unit, Aligarh Muslim University, Aligarh, India (Approval ID: 332/CPCSEA). All the animal experiments were performed according to the National Regulatory Guidelines issued by the Committee for the Purpose of Control and Supervision of Experiments on Animals (CPCSEA), Government of India.

### Preparation of orange juice

Edible juicy part (30 g) of orange was chopped into small pieces and blended in a food grinder^37^. Briefly, (30 g) of orange pulp was mixed with 100 ml of double distilled water then it was grounded in mixer. The obtained extract was boiled for several minutes till its volume was reduced to 1/3 of initial volume. The boiled extract was filtered through Whatman filter and the filtrate was stored at −20 °C till further use.

### Preparation of Nano-drug formulation employing orange juice (OJ)

The synthesis of 5-FU nano-drug formulation was executed employing orange fruit extract. The increasing volumes [1–5 ml of 30% w/v stock solution (Stock solution: 30 g pulp in 100 ml of water)] of extract were added to 5 ml of 1 mM solution of 5-FU and the volume was made up to 10 ml by deionized water. The mixture was incubated for a given time period at 25 °C, followed by centrifugation at 1,00,000 g for 2 hr to pellet the 5-FU nanoparticles. The pellet obtained was lyophillized to obtain free flowing powder. The nanoparticles were suspended in 1 ml of PBS (10 mM phosphate, 150 mM NaCl, pH 7.4) and further characterized by various spectrophotometric and microscopic techniques.

### Characterization of 5-FU NPs

#### Transmission electron microscopy

A JEOL Transmission Electron Microscope (JEOL, Tokyo, Japan) was used for Imaging of in-house synthesized 5-FU Nanoparticle. Briefly, samples were prepared following a published method by drying a drop of synthesized NPs on a carbon coated copper grid. TEM micrographs were acquired with an accelerating voltage of 100/120 kV.

#### Particle size distribution and Zeta-potential

Particle size distribution of the synthesized NPs was analyzed using dynamic light scattering on DynaPro-TC-04 DLS instrument (Protein Solutions, Wyatt Technology, Santa Barbara, CA) the size distribution of as-synthesized NPs was also observed by Nanophox particle size analyzer (Sympatec GmbH, Clausthal-Zellerfeld, Germany). To evaluate the stability and surface charge of the as synthesized 5-FU NPs, zeta potential was determined using DTS software (Malvern Instruments, Worcestershire, UK) based on M3-PALS technology following a method standardized in our laboratory^38^. 5-FU NPs were suspended in phosphate buffer (10 mM, pH 7.4) and thoroughly mixed in a cyclo-mixer for 1 min and used for zeta sizing as well as surface charge determination.

#### UV-spectrophotometric analysis

Biomimetically synthesized 5-FU NPs were analyzed for surface plasmon resonance by employing double beam UV-Visible spectrophotometer (Perkin Elmer, Boston, MA) and scanned in 180–700 nm wavelength range using 10 mm optical path length quartz cuvettes.

#### Atomic Force Microscope (AFM) Imaging

AFM was performed by using Perkin-Elmer digital AFM microscope equipped with a Nano-scope controller following published protocol^39^. 5-FU NPs were suspended in 1 ml (10 mM PBS, pH 7.4) of followed by brief ultra-sonication to agitate particles in a solution in order to prevent agglomeration of NPs. Subsequently, a drop of the diluted 5-FU NPs was put onto the Si (III) disc for AFM imaging.

#### Differential scanning calorimetry (DSC)

DSC study was carried out on dried samples of pure 5-FU (control) and formulation containing 5-FU. Briefly, 3–5 mg dried powder was placed in an aluminium pan. Empty aluminium pan was used as reference. The sample pan and reference pan were heated at constant flow rate of 10 ^o^C/min within the temperature range of 10–350 ^o^C (Simultaneous Thermal Analyser (STA) 8000, Perkin Elmer, USA). Nitrogen gas was continuously purged (Flow rate = 20 ml/min) throughout the experiment. The data obtained was smoothened and corrected for baseline using Origin Pro (64 bit, Sr3, b275, OriginLab Corporation, Northampton, MA01060, USA)^19,20^.

#### Evaluation of *in-vitro* Nano-drug release kinetics

Release kinetics of 5-FU NPs, was examined by dispensing multiple samples of the formulation into various micro vials following the protocol as standardized in our lab. To each vial, 1.0 ml of release medium was dispensed along with 0.01% of sodium azide to prevent microbial growth. At stipulated time period, an aliquot (40 µl) of supernatant was removed after centrifugation at 1 × 10^5^ for 10 minutes at 25 °C. The aliquots were replenished with fresh buffer to maintain constant volume of the suspension. HPLC of 5-FU was performed following a published method that was modified in our lab^40^. Briefly, chromatographic separation was carried out using symmetry C18, 5 µm (3.9 × 150 mm) column. Mobile phase consisted of 40 mM phosphate buffer, pH 7.0 with 10% (w/v) potassium hydroxide. Flow rate was kept 1.0 ml/min and detection wavelength was 260 nm. At every time point, 20 µl of sample was injected and Absorbance was recorded. Drug concentration of the sample was calculated from the calibration curve prepared using pure drug mixed in release medium.

#### Tumor induction

Male BALB/c mice in the resting phase of hair cycle were included for the induction of skin fibrosarcoma. The animals were shaved in the dorsal portion over an area of 2–3 cm^2^ employing electric clippers followed by the exposure to DMBA (52 µg in 200 µl acetone) that was applied topically three times a week for 12 weeks. At the end of 12 weeks, the mice which had ~120 mm^3^ tumor size were used in the study.

#### Treatment schedule

To assess the anticancer efficacy of 5-FU NPs, animals were divided in four various groups each consisting of 25 animals. Group I consisted of DMBA exposed BALB/c mice (having papilloma growth) that were subsequently treated with carrier only and served as a control group. The remaining three groups consisted of animals that were earlier exposed to DMBA and had papilloma growth on their skin. Group II was treated with OJ along with cream base (5% v/v of OJ stock solution in cream base). The tumor bearing animals belonging to group III were treated with microcrystals of parent 5-FU drug along with cream base (5% w/v of microcrystal of parent 5-FU drug in cream base). Group IV was treated with 5-FU NPs along with cream base (5% w/v of 5-FU NPs in cream base).

#### Determination of tumor volume

The measurement of tumor diameter of the treated groups of animals was performed by employing Vernier Caliper and the tumor size was calculated by the following formula:

$${\rm{V}}={{\rm{D}}}^{{\rm{2}}}\times {{\rm{d}}}^{{\rm{2}}}\times {\rm{\pi }}/{\rm{6}}$$

where V is the measure of tumor volume, D is the measure of biggest dimension of the tumor and d is the measure of smallest dimension of the tumor. The tumor tissues were excised from the animals and underwent entire skin examination.

#### Histopathological studies

To assess efficacy of 5-FU NPs against the tumor development, skin tumor tissues were sliced from three mice of each group and fixed in 10% formaldehyde solution, dehydrated in ascending grades of ethyl alcohol, cleared in xylol and mounted in molten paraplast at 58–62 °C. The finely cut thin sections were stained with hematoxylin and eosin and examined for any morphological change under an Olympus BX40 microscope (PA, USA).

#### Isolation of nuclear fraction from the tumor tissues

The tumor tissues were aseptically excised from skin of animals belonging to various treated groups. Briefly, the tissues were homogenized in a REMI homogenizer (REMI Laboratory Instruments, New Delhi, India) to prepare nuclear fraction in the presence of protease inhibitor cocktail and PMSF following published method as modified in our laboratory^41^.

#### Expression level of various apoptotic markers upon treatment with 5-FU Nano-particles

Western blot was performed for the analysis of expression of various apoptotic markers like p53wt, p53mut, Bcl-2 and Bax in tumor tissues of the treated groups^42^. Before resolving expressed protein employing SDS-PAGE, protein content of each sample was estimated using a Bicinchoninic Acid solution kit (Sigma-Aldrich, Co., USA)^43^. Briefly protein (30 µg/lane) was loaded and resolved by electrophoresis on 10% sodium dodecyl sulfate-polyacrylamide gel and transferred onto PVDF membrane. After blocking in 5% non-fat dry milk prepared in phosphate-buffered saline (PBS) with Tween-20 (PBS-T), the membrane was washed three times with PBST and incubated for 2 hrs at 37 °C with rabbit anti-mouse p53wt, rabbit anti-mouse p53mut, rabbit anti-mouse Bax and rabbit anti-mouse Bcl-2 primary antibodies. After incubation and stipulated washing steps, the membrane was further incubated with HRP conjugated goat-anti-mouse secondary antibody (1:5000) for 1 hr at 37 °C. Blots were developed with the ECL (Enhanced chemi-luminescence) detection system (Bio-Rad). The pixel density of the bands was quantified using GS-800 Calibrated Imaging Densitometer (Bio Rad, India).

#### Assessment of apoptosis potential of 5-FU NPs employing Fluorescence microscopy

The DMBA induced fibrosarcoma tissues were removed from the treated mice of respective experimental groups. Briefly, excised tissue was cut into small pieces with scalpel, dissociated with the frosted slides and suspended in the petri-dish containing 1 ml of collagenase P (3 mg/ml) dissolved in 15 ml of RPMI media containing 10% FBS. Incubated at 37 °C with gentle shaking and after 3 hr of incubation passed through cell strainer. Single cell suspension of 1–2 × 10^6^ cells was prepared by pelleting down cells at 1600 × g for 5 min at 4 °C. Cell suspension was fixed in 1% paraformaldehyde in PBS (pH 7.4) and placed on ice for 30 min. Cells were settled down at 300 × g for 5 min and washed with 5 ml of PBS (pH 7.4). Pelleted cells were re-constituted in 50 µl PBS (pH 7.4) and fixed in 2 ml of 70% chilled ethanol. Suspension was again washed twice with 1 ml of wash buffer and pelleted cells were re-constituted in 50 μl of DNA labeling solution and incubated for 60 min at 37 °C. After incubation, cells were washed and re-suspended in FITC-conjugated anti-bromodeoxyuridine (BrdU) antibody at 25 °C for 30 min. The cell suspension was washed and further dissolved in 1 ml of PBS and 50 μg/ml PI/RNase was added to antibody-labeled cells, which were further incubated at 37 °C for 30 min. Stained cells were finally analyzed by Zeiss, Axiocam Imager MRM M2(Thornwood, NY United States)^44^.

#### Determination of caspase-9 level in apoptotic cells post 5-FU Nanoparticle treatment

Single cell suspensions of the excised tissue from the various respective group were fixed with 1% paraformaldehyde plus 0.19% picric acid in PBS (pH 7.4) for 1 hour at RT. Subsequently, the fixed cells were treated with permeabilizing solution containing 0.1% SDS in PBS incubated for 10 min at RT and stained with a primary antibody (caspase-9 p35 antibody) that specifically binds with the p35 subunit of caspase-9 and further incubated with a goat anti-rabbit IgG-FITC conjugated secondary antibody (Sigma-Aldrich, USA)^45^. Fluorescence imaging was conducted using a Zeiss, Axiocam Imager MRM M2(Thornwood, NY United States).

#### Apoptosis detection employing FITC labeled annexin-V binding analysis

To evaluate the apoptotic potential of 5-FU NPs, skin samples from animals belonging to various treated groups, were collected. Single cell suspension was prepared as described elsewhere and analyzed with commercial FITC labelled Annexin-V (BD Pharmingen) according to the manufacturer’s instructions. The cells were incubated with 5 µL FITC Annexin-V. Apoptosis was analyzed by Annexin-V FITC and PI staining by flow cytometry. AnnexinV−, PI− represent live cells, annexinV+, PI− represent early apoptotic cells, and annexin V+, PI+ cells represent late apoptotic cells. The total cell undergoing apoptosis can be represented as annexin V+. Cells were gently vortexed and incubated at room temperature for 15 min. Finally, 400 µL of 1X binding buffer was added and cells were analyzed using BD FACSVERSE flow cytometer, (BD Biosciences, United States)^46^.

### Statistical analysis

Results were expressed as the mean ± SEM and data were analysed by means of one way analysis of variance (ANOVA) and two-way ANOVA to assess the differences among various groups. Statistical calculations were performed with the help of Graph-Pad prism version 6.0, Graphpad software Inc San Diego, California, USA. Significance was indicated as *** for P < 0.001; ** for P < 0.01 and * for P < 0.05. Student T-test was used to study difference in biochemical parameters between different treatment groups. The difference with P < 0.05 considered to be significant. Kaplan-Mier Curve was used for survival analysis of the treated groups.

## Supplementary information

Bio-mediated synthesis of 5-FU based nanoparticles employing orange fruit juice: a novel drug delivery system to treat skin fibrosarcoma in model animals
