# Supplementary material for: Bio-mediated synthesis of 5-FU based nanoparticles employing orange fruit juice: a novel drug delivery system to treat skin fibrosarcoma in model animals
Source: Sci Rep. 2019 Aug 23;9:12288. doi: 10.1038/s41598-019-48180-7 (PMC6707153; doi:10.1038/s41598-019-48180-7)
Supplement: Supplementary file 1 — Bio-mediated synthesis of 5-FU based nanoparticles employing orange fruit juice: a novel drug delivery system to treat skin fibrosarcoma in model animals [file 41598_2019_48180_MOESM1_ESM.docx]

**Supplementary Materials for**

**Title:** Bio-mediated synthesis of 5-FU based nanoparticles employing orange fruit juice: a novel drug delivery system to treat skin fibrosarcoma in model animals

**Authors:** Owais Mohammad1*, Syed Mohd Faisal1, Nadeem Ahmad2, Mohd. Ahmar Rauf1, Mohd Saad Umar1, Anzar Abdul Mujeeb1, Piyush Pachauri1, Anees Ahmed3, Mohammad Kashif1, Mohammad Ajmal4, Swaleha Zubair5*

**Affiliations:**

1Interdisciplinary Biotechnology Unit, AMU. Aligarh, INDIA

2University of Jeddah, Jeddah, KSA

3 National Institute of Immunology, New Delhi, INDIA

4 Jawaharlal Nehru Medical College, AMU. Aligarh, INDIA

5Womens College, AMU, Aligarh, INDIA

***Corresponding authors:**

[owais_lakhnawi@yahoo.com](mailto:owais_lakhnawi@yahoo.com); Owais Mohammad of Interdisciplinary Biotechnology Unit, Aligarh Muslim University, Aligarh, UP-202002, INDIA

[swalehazubair@yahoo.com](mailto:swalehazubair@yahoo.com); Swaleha Zubair, Women’s College, Aligarh Muslim University, Aligarh, UP-202002, INDIA.

**This file includes:**

Supplementary Text

Figures S1, S2, S3 ,S4 and S5

**Methods employed in characterization studies of 5-FU Nps**

**Transmission Electron Microscopy**

A JEOL Transmission Electron Microscope (JEOL, Tokyo, Japan) was used for imaging of in- house synthesized 5-FU Nanoparticles. Briefly, samples were prepared following a published method by drying a drop of synthesized NPs on a carbon coated copper grid. TEM micrographs were acquired with an accelerating voltage of 100/120 kV.

**Atomic Force Microscope (AFM) Imaging**

AFM imaging was performed by using Perkin-Elmer digital AFM microscope equipped with a Nano-scope controller following published protocol39. 5-FU NPs were suspended in1ml of deionized water followed by brief ultra-sonication (to agitate particles in a solution), in order to prevent agglomeration of NPs, and then a drop of the diluted 5-FU NPs was put onto the Si (III) disc for AFM imaging.

**GC-MS analysis**

The filtered extract of orange juice (30 g pulp in 100 ml of deionized water) was lyophilized and stored at +4 C until analysed. The sample was analysed by gas chromatography/mass spectrophotometry (GC/MS) techniques using an Agilent 5975 GC-MSD system coupled to an

Agilent 7890 A GC (Agilent Technologies Inc., Santa Clara, CA). HP-Innowax FSC column

(60 × 0.25 mm, 0.25 m ﬁlm thickness) was used with helium (purity 99.99%) as carrier gas

(1.2 mL/min). GC oven temperature was kept at 60 C for 10 min and programmed to 220 C

at a rate of 4 ◦C/min, and kept constant at 220 C for 10 min and then programmed to 240 C

at a rate of 1/min. The split ratio was used at 40:1. The injector temperature was adjusted to

250 C and mass spectra were recorded at 70 eV. Mass range was adjusted from 35 to 450 m/z. In order to obtain the same elution order with GC/MS, simultaneous triplicate injections were

done by using the same column and same operational conditions.

**HPLC analysis**

Liquid chromatography of orange juice was performed using an HPLC system model Agilent series 1100 (Agilent, Waldbronn, Germany) including a quaternary pump (Agilent G 1311A), a vacuum degasser (Agilent G 1322A) and an autosampler (Agilent G 1313A). The HPLC

system was directly coupled to a triple quadrupole mass spectrometer (model Sciex API 2000, Applied Biosystems, Langen, Germany) equipped with an electrospray ionisation (ESI) interface. The column was eluted with methanol. The parameters used were at the time of run Interval @ 500 msec, number of points 7201, run time 60 min and Intensity multiplier for all the wavelength was 0.001 mV. Prior to the next injection, the column was equilibrated for 12 min, resulting in a total run time of 72 min.

**In vitro anticancer efficacy of 5-FU NPs against A253 epidermal cells. MTT assay**

The effect of both free as well as 5-FU NPs on viability of epidermoid cells was determined by MTT assay. MTT (Thiazolyl Blue Tetrazolium Bromide), a yellow dye, gets converted into formazan by the mitochondrial dehydrogenase enzyme. MTT assay on epidermoid cells (A253 cell line) as performed as described before (Mosmann *et al* 1983). Briefly, exponentially growing cells, were dispensed into 96-well plates, at density of 1.0 × 105 cells per well and cultured for 48 hours. Subsequently, cells were incubated with varying concentration of 5-FU NPs (15-30 µg/ml NPs). The free drug (microcrystalline 5-FU ) and OJ were taken as control. After 48 hours of incubation, MTT solution (5 mg/mL) was added to each well. The precipitated formazan, formed after 4 hours incubation, was dissolved in 100 μL DMSO, and absorbance was measured using an enzyme-linked immunosorbent assay (ELISA) reader (Thermo Molecular Devices Co, Union City, NJ, USA), at 570 nm. Cell viability ratio was calculated using the following equation:

Cell viability ratio (%) = ODtreated/ ODcontrol × 100% [1]

**Effect of 5-FU NPs on the expression of various cell cycle check point regulating factors**

Epidermoid cells (A253), treated with various forms of 5-FU for 12 hrs at their IC50 concentration, were pelleted and lysed using Cell Lysis Reagent (Sigma, St. Louis, MO, USA) in the presence of Na-orthovanadate, Na-fluoride, and protease inhibitor cocktail. The total protein content was measured by the BCA method **[2]** using bovine serum albumin as the standard. The cell lysate (30 µg protein/lane) was resolved by the SDS-polyacrylamide gel electrophoresis and transferred on to a nitrocellulose membrane. The membrane was blocked with 3% BSA in PBST for 1.5 h at room temperature and probed with anti-human primary antibodies (1:1,000) against p53, Bcl2 and Bax (BD biosciences, USA) for 90 min at room temperature followed by overnight incubation at 4°C. The membrane was further incubated for

1.5 h at room temperature with secondary antibody conjugated to horseradish peroxidase (BD, Biosciences). The protein bands were visualized by enhanced chemiluminescence (Bio-Rad) and densitometry was done utilizing the Bio-Rad (GS800). Expression of β-actin was taken as a control standard3,4.

**Hoechst staining**

Apoptotic nuclear morphology of 5-FU treated cells was evaluated using Hoechst 33342 (BD BIO, MO, USA). The cells were exposed to both free as well as 5-FU NPs at their IC50 values. Subsequently, the cells were fixed with 4% paraformaldehyde at room temperature for 30 min. The treated cells were washed with PBS followed by staining with 2μg/ml Hoechst 33342 at

37 °C for 30 min. The cells were washed and their morphology was observed at 40X;

employing Zeiss fluorescence microscope2.

**Annexin-V-FITC/PI staining**

Annexin-V-FITC/PI staining was performed employing Annexin V-FITC apoptosis detection kit (Sigma, St Louis, MO, USA) as per the manufacturer’s protocol. Briefly, the cells were harvested by trypsinization, washed with PBS and re-suspended in binding buffer (10 mM HEPES/NaOH, pH 7.5 containing 140 mM NaCl and 2.5 mM CaCl2) at a concentration of 106 cells/ml. The cells were incubated with both free as well as 5-FU NPs at the concentration of

25 µg/ml for 12 hours. Annexin V-FITC (5 µl) and propidium iodide (10 µl) was added to 500

µl of cell suspension and incubated for 30 min in the dark conditions at RT. The cells were immediately analyzed by flow cytometry (FACS Canto TM II, BD BioSciences, San Jose, CA, USA). Dot plots were analyzed by FACS Diva 6.1.2 software. FACS plots show apoptosis in samples treated with increasing concentration nanoparticles. Apoptosis was analyzed by Annexin-V FITC and PI staining by flow cytometry. Annexin-V-, PI- cells are live cells, Annexin-V+, PI- cells correspond to early apoptotic cells, and Annexin-V+, PI+ cells represent late apoptotic cells**5.**

**Apoptosis Assay by Acridine Orange/Ethidium Bromide Staining.**

Epidermoid cells treated with both free 5-FU as well as 5-FU NPs at 20μg/ml for various time periods. An aliquot (100μl) of treated cells ( ~ 1.25 × 106 per mL) was incubated with 1 μl of acridine orange/ethidium bromide (one part each of 100 μg/ml acridine orange and 100 μg/ml ethidium bromide in PBS). A 10 μl aliquot of the gently mixed suspension was placed on microscope slides, covered with glass slips, and examined under Zeiss microscope connected

to a digital imaging system. Acridine orange is a vital dye that stains both live and dead cells, whereas ethidium bromide stains only those cells that have lost their membrane integrity. Live cells attained uniform green fluorescence and can be distinguished from apoptotic cells as they acquire yellow to orange coloration depending on the degree of loss of membrane integrity due to co-staining with ethidium bromide6.

**RESULTS**

**Characterization of 5-FU parent material and 5-FU NPs by employing UV, TEM and AFM analysis.** We extensively characterized both as-synthesized 5-FU NPs and 5FU parent drug using various spectrophotometric techniques as well as AFM and electron microscopy. The TEM analysis revealed micro-meter size dimensions of parent 5 FU microcrystals (Figure S1A). Incubation of 5-FU parent drug in the physiological PBS for 24 hrs resulted in slight reduction in size when observed at micro-meter scale (Figure S1B). We established formation of isomorphic hexagonal 5-FU NPs with nano size dimension upon incubation of 5 FU microcrystal with OJ for stipulated time period (Figure S1C). The UV spectrophotometric study suggests that free 5-FU microcrystals absorbs maximally at 271 nm (Figure S1D)**.** Interaction of 5-FU with OJ (for 10 hrs) resulted in 5 FU NPs showing significant quenching in absorbance of characteristic peaks belonging to parent 5-FU (Figure S1D). Interestingly, resurrection in absorption peaks in 5 FU NPs was observed that were formed upon longer incubation (18 hr duration) of parent 5 FU with OJ (Figure S1D). A myriad of hexagonal shaped 5-FU NPs was observed upon 2D AFM microscopy as shown in Figure S1E. The size dimensions of as-synthesized 5-FU NPs was in the range of 30±5 nm as confirmed by 3D AFM Image (Figure S1F).

**Cell Viability (MTT) assay**

Figure S2A shows effect of increasing concentrations of both free as well as 5-FU NPs on epidermoid cells viability. The treatment with 5-FU NPs for 12 hours markedly inhibited epidermoid cells. The survival of the epidermoid cells was inversely correlated with concentration of the NPs, indicating a dose-dependent inhibitory effect. The IC50 of 5-FU NPs was ~18 μg/ml, while the IC50 of free 5-FU drug was ~ 32 μg/ml (P value < 0.01). **(Figure S2A).**

**Effect of 5-FU treatment on expression of apoptotis regulating factors employing Western blot analysis**

The anticancer efficacy of various 5-FU formulations was assessed using Western blot profiling of crucial apoptotic molecules in various experimental groups. As shown in **Figure S2B,** 5-FU NPs had more prominent effect on expression of pro-apoptotic molecules as compared to free-form of 5-FU. OJ induced similar expression level of apoptotic factors to that of control untreated group.

**Hoechst 33342 DYE staining establishes 5FU-NPs mediated apoptosis**

The apoptotic potential of 5-FU NPs was ascertained employing Hoechst 33342 staining. The treated cells were examined for nuclear morphology. The cells from control group had round and homogenously stained nuclei, while cells exposed to various 5-FU formulations showed marked DNA condensation and the presence of apoptotic bodies. **(Figure S2C).**

**PI and ANNEXIN V Binding assay employing FACS analysis**

Flow cytometry analysis was conducted using Annexin-V-FITC/PI staining to analyze the apoptosis induced by 5-FU NPs. Epidermoid cells were treated with 5-FU NPs for 12 h. The cells from control group did not take Annexin V-FITC and propidium iodide stain. However, the cells treated with a free 5-FU had shown around 30 % while NPs induced 34% apoptotic cells respectively **(Figure S2D).**

**Dual AO/EtBr Staining to Detect Apoptosis**

The apoptotic potential of 5-FU NPs was further established by dual AO/EtBr staining. The control group demonstrated green fluorescence only. However, treatment with free 5-FU drug and 5-FU-NPs resulted in early-stage apoptotic cells. The early sage of apoptotic cells were marked by crescent-shaped or granular yellow-green acridine orange nuclear staining. Late- stage apoptotic cells were marked with focused and lopsidedly localized orange nuclear ethidium bromide staining. Necrotic cells had undergone increase in volume and showed uneven orange-red fluorescence at their periphery. 5-FU NPs treated cells appeared to be in the process of disintegration **(Figure S2E).**

**Modulation of apoptosis-related factors in skin fibrosarcoma upon treatment with 5-FU NPs suspended in normal saline**. We assessed efficacy of as-synthesized 5-FU NPs (suspended in normal saline) against skin fibrosarcoma in experimental animals. Briefly, we determined efficacy of 5-FU NPs in terms of survival rate of experimental animals harbouring

skin fibrosarcoma (Figure S5A). We also assessed potential of 5FU NPs to regress skin tumors (papilloma) in experimental animals (Figure S5B). Figure S5C shows modulation of various apoptotic factors viz. *Bax*, Bcl2 and P53 in skin fibrosarcoma of experimental animals upon treatment with 5-FU NPs (in normal saline).

**Statistical Analysis**

Results were expressed as the mean ± SD. The data were analyzed by means of one-way analysis of variance (ANOVA) and two-way ANOVA to assess the differences among various groups. Statistical calculations were performed with the help of Graph-Pad prism version 6.0, GraphPad software Inc San Diego, California, USA.

**REFERENCES**

1. Mosmann, T. Rapid colorimetric assay for cellular growth and survival: Application to proliferation and cytotoxicity assays. Journal of Immunological Methods 65, 55–63 (1983).

2. Sharma, V., Anderson, D. & Dhawan, A. Zinc oxide nanoparticles induce oxidative DNA damage and ROS-triggered mitochondria-mediated apoptosis in human liver cells (HepG2). *Apoptosis* **17,** 852–870 (2012).

3. Walker, J. M. in *The Protein Protocols Handbook* (ed. Walker, J. M.) 11–14 (Humana

Press, 2002). doi:10.1385/1-59259-169-8:1

4. Green, D. R. & Reed, J. C. Mitochondria, and Apoptosis. Science 281, 1309 LP-1312 (1998).

5. Biosciences, B. D. Detection of Apoptosis Using the BD Annexin V FITC Assay on the BD FACS VerseTM System. (2011).

6. Liu, K., Liu, P., Liu, R. & Wu, X. Dual AO/EB Staining to Detect Apoptosis in Osteosarcoma Cells Compared with Flow Cytometry. Medical Science Monitor Basic Research 21, 15–20 (2015).

**LEGENDS TO SUPPLEMENTORY FIGURES**

**Figure-S1. Characterization of 5-FU parent material and 5-FU NPs by employing UV, TEM and AFM analysis.** Characterization of the synthesized 5-FU NPs and 5FU parent drug. **(a)** The TEM analysis revealed microcrystals of parent drug, **(b)** upon incubation of 5-FU parent drug in the PBS for 24 hrs there was slight reduction in size when observed at micro-meter scale. **(c)** Formation of isomorphic hexagonal 5-FU NPs with nano size dimension. **(d)** The incubation of free 5 FU microcrystals with OJ (0 hr incubation time period) absorbs maximally at 271 nm as revealed by UV absorption spectroscopy**.** Interaction of 5-FU with OJ for 10 hrs resulted in significant quenching in absorbance of characteristic peaks belonging to 5-FU. There was resurrection in absorption peaks observed upon incubation of 5-FU NPs with OJ for longer (18 hr) duration. **(e)** A myriad of hexagonal shaped 5-FU NPs was observed upon 2D AFM microscopy. **(f)** The size of as-synthesized 5-FU NPs was in the range of 30±5 nm as confirmed by 3D AFM Image.

**Figure-S2. GC-MS analysis of Orange fruit juice extract (stock solution).** To identify the orange fruit juice extract component, we performed GC-MS analysis. The Identiﬁcation of various constituents was executed on the basis of retention index (RI). Rigorous identiﬁcation analysis was carried out by comparison of their mass spectra with those from NIST 05 and Wiley 8th version and home-made MS library built up from pure substances and components of known EOs, as well as by comparison of their RIs with literature values. Spectra and constituent present in the extract is represented in the Figure-S2.

**Figure-S3. HPLC analysis of Orange fruit juice extract (stock solution) at different wavelengths.** LC chromatogram of orange fruit juice extract at 280 nm (a), 320 nm (b) and 360 nm (c).

**Figure-S4A. Effect of 5-FU NPs on the viability of epidermoid cells (A253).** Epidermoid cells were treated with varying concentrations of 5-FU NPs for 12 hrs. Post treatment, cell viability was determined using MTT assay, as described in Materials and methods. Data represent mean ± standard deviation of three identical experiments performed in the triplicate.

**Figure-S4B**. **Modulation of apoptosis-related factors in epidermoid cells as revealed by Western blot analysis**: The effect of 5-FU NP on expression level of various apoptotic factors viz. *Bax*, Bcl2 and P53 in A253 cells was established by western blot analysis using antibodies specific for various apoptotic factors. Beta-actin was used as internal control to normalize the data; (B) Relative quantification of protein expression levels. Statistical significance was calculated using unpaired student’s t-test. p≤0.05 is considered statistically significant (p≤0.01; Group 4 vs Group 1, p≤0.01; Group 3 vs group 1, p≤0.01; group 2 vs group 1 ). Data represented as mean ± Standard Deviation.

**Figure-S4C. Fluorescence micrographs showing effect of 5-FU NPs as revealed by Hoechst 33342 staining**. 5-FU NPs induced apoptosis in A253 cells. The apoptosis was marked by condensed or fragmented nuclei (indicated by arrows) after exposure of 12 hrs. (A) control untreated cells (B). Cells treated with 5-FU drug only (C) Cells treated with 5-FU NPs.

**Figure-S4D**. **FACS analysis of A253 cells treated with various 5-FU formulations.** 5-FU formulation induced apoptosis in A253 epidermoid cells. Flow cytometric analysis of Annexin V-FITC/PI stained cells. Data represent mean ± SD of three experiments. Representative dot plots of three independent experiments are presented. [P value ** Control v/s 15 µg/ml NPs P≤

0.01; control v/s 30 µg/ml NPs P≤ 0.01; 15 µg/m NPs l v/s 30 µg/ml NPs P≤ 0.01].

**Figure-S4E. 5-FU NPs induces apoptosis in A253 epidermoid cells as revealed by AO- EtBr assay.** Normal cells show circular nucleus uniformly distributed in the center of the cell. The observed yellow-green fluorescence corresponds to acridine orange (AO) staining, while orange fluorescence corresponds to ethidium bromide staining of nucleus belonging to groups treated with various 5-FU formulations (Magnification 20X).

**Figure-S5. Modulation of apoptosis-related factors in skin fibrosarcoma upon treatment with 5-FU NPs**. The efficacy of as-synthesized 5-FU NPs (suspended in normal saline) against skin fibrosarcoma. **(A)** Efficacy of 5-FU NPs in terms of survival rate of experimental animals with fibrosarcoma. **(B)** Tumor (papilloma) regression efficiency of 5-FU NPs (suspended normal saline) against skin fibrosarcoma in experimental animals. **(C)** Modulation of various apoptotic factors viz. *Bax*, Bcl2 and P53 in skin fibrosarcoma of experimental animals upon treatment with 5-FU NPs (in normal saline).


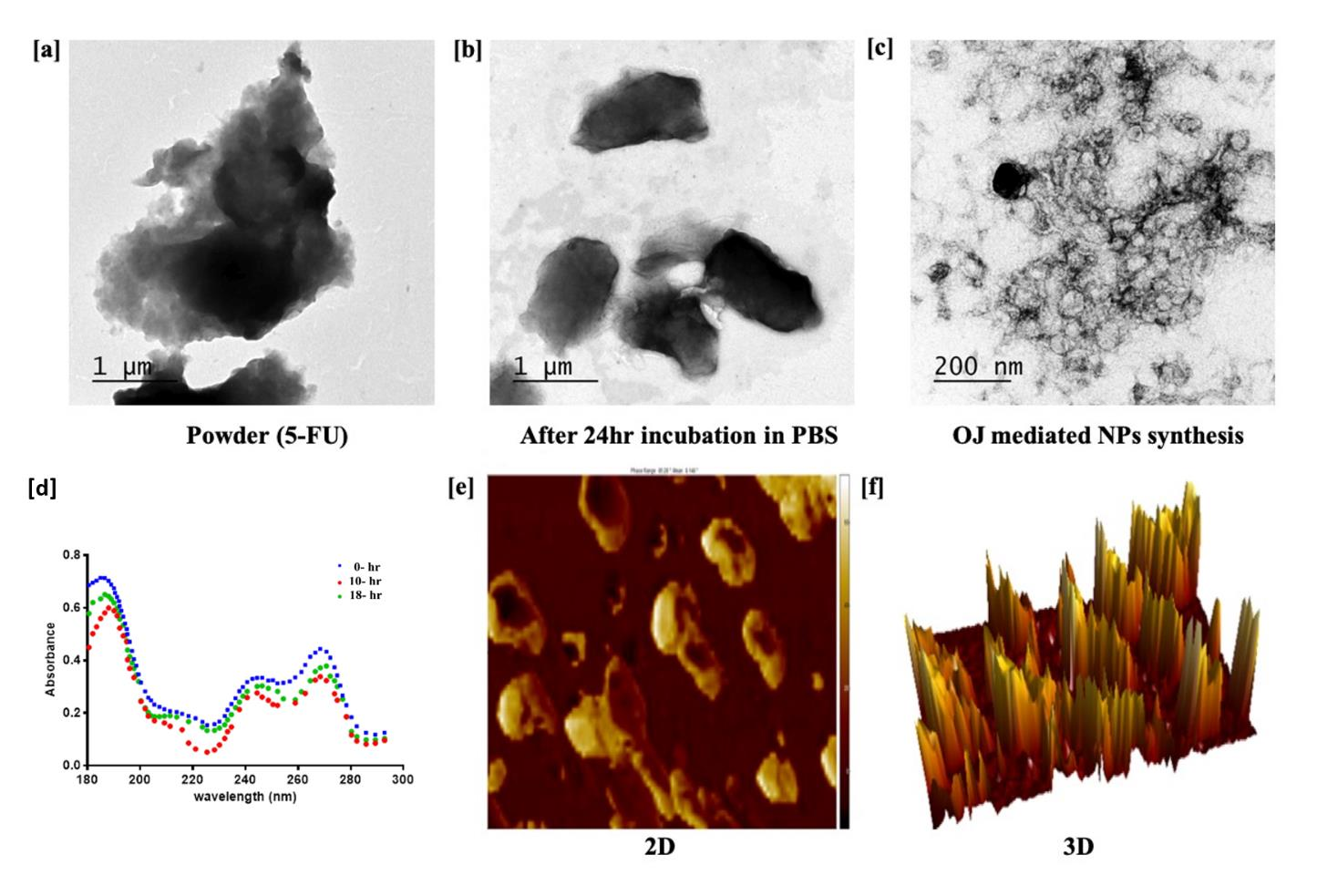


**Figure-S1**


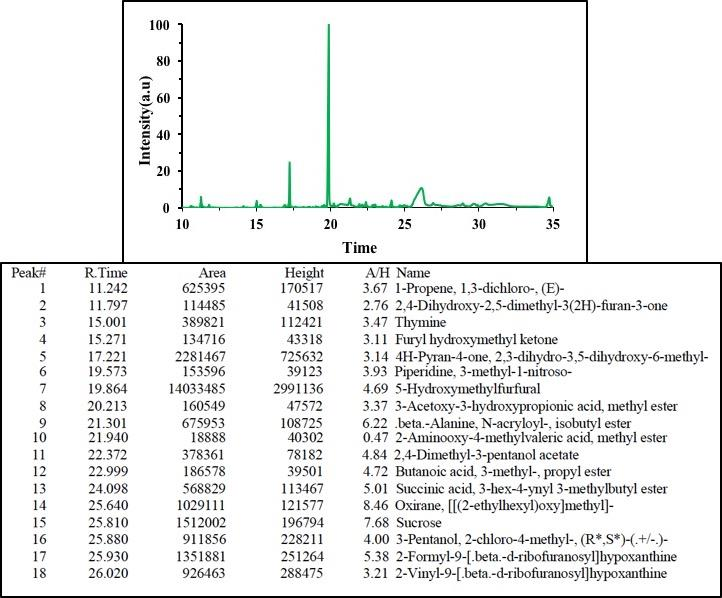


**Figure-S2**


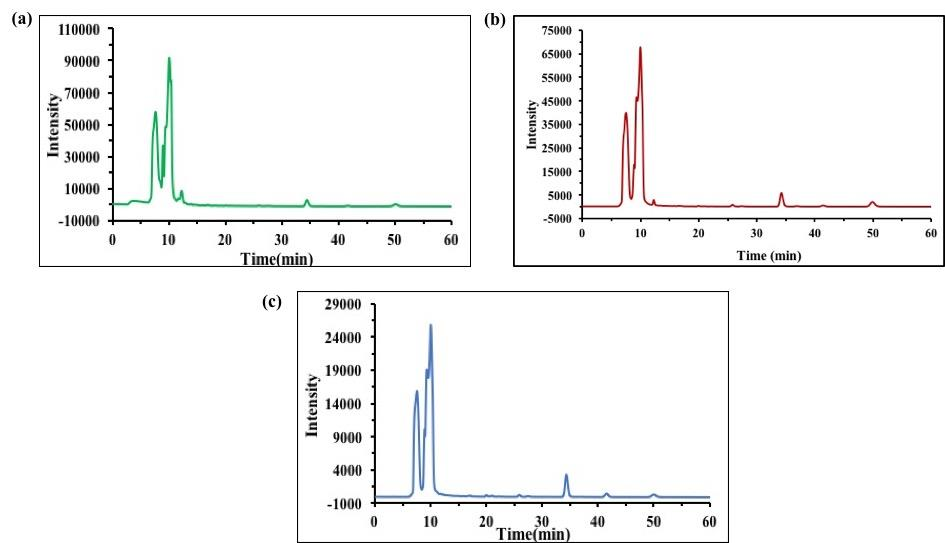


**Figure-S3**


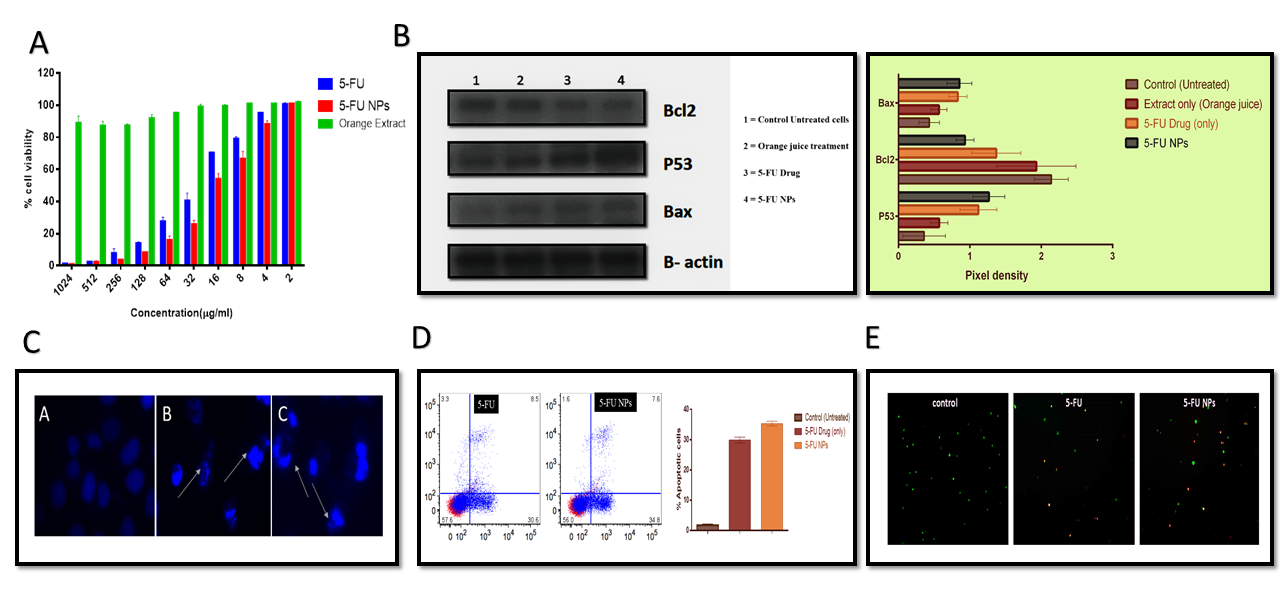


**Figure-S4**


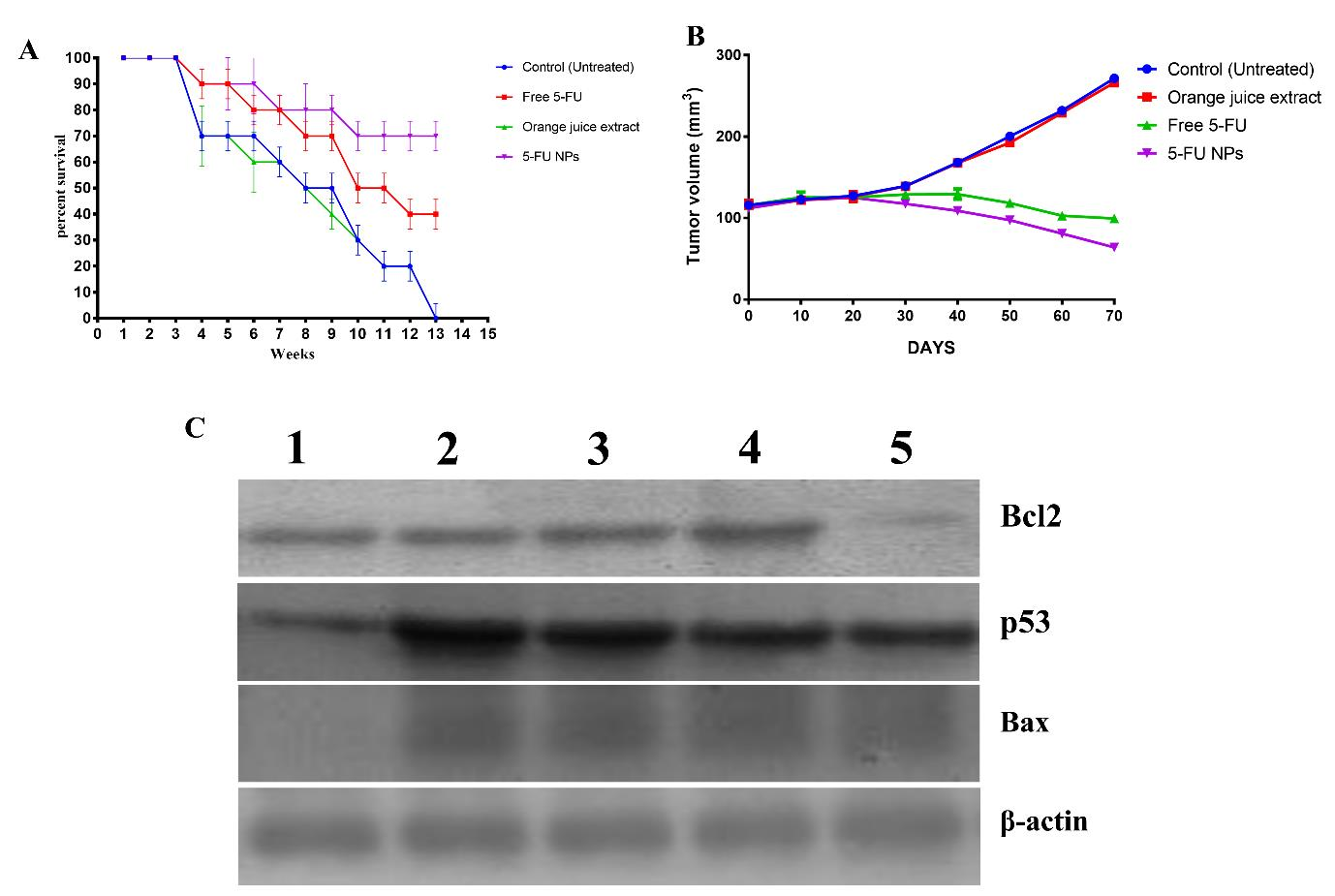


**Figure-S5**
